# A parapoxviral virion protein inhibits NF-κB signaling early in infection

**DOI:** 10.1371/journal.ppat.1006561

**Published:** 2017-08-07

**Authors:** Sushil Khatiwada, Gustavo Delhon, Ponnuraj Nagendraprabhu, Sabal Chaulagain, Shuhong Luo, Diego G. Diel, Eduardo F. Flores, Daniel L. Rock

**Affiliations:** 1 Department of Pathobiology, College of Veterinary Medicine, University of Illinois at Urbana-Champaign, Urbana, Illinois, United States of America; 2 School of Veterinary Medicine and Biomedical Science, Nebraska Center for Virology, University of Nebraska-Lincoln, Lincoln, Nebraska, United States of America; 3 Departamento de Medicina Veterinária Preventiva, Centro de Ciências Rurais, Universidade Federal de Santa Maria, Santa Maria, Rio Grande do Sul, Brazil; University of Utah, UNITED STATES

## Abstract

Poxviruses have evolved unique proteins and mechanisms to counteract the nuclear factor κB (NF-κB) signaling pathway, which is an essential regulatory pathway of host innate immune responses. Here, we describe a NF-κB inhibitory virion protein of orf virus (ORFV), ORFV073, which functions very early in infected cells. Infection with ORFV073 gene deletion virus (OV-IA82Δ073) led to increased accumulation of NF-κB essential modulator (NEMO), marked phosphorylation of IκB kinase (IKK) subunits IKKα and IKKβ, IκBα and NF-κB subunit p65 (NF-κB-p65), and to early nuclear translocation of NF-κB-p65 in virus-infected cells (≤ 30 min post infection). Expression of ORFV073 alone was sufficient to inhibit TNFα induced activation of the NF-κB signaling in uninfected cells. Consistent with observed inhibition of IKK complex activation, ORFV073 interacted with the regulatory subunit of the IKK complex NEMO. Infection of sheep with OV-IA82Δ073 led to virus attenuation, indicating that ORFV073 is a virulence determinant in the natural host. Notably, ORFV073 represents the first poxviral virion-associated NF-κB inhibitor described, highlighting the significance of viral inhibition of NF-κB signaling very early in infection.

## Author summary

Successful infection of the host by poxviruses relies on control of innate immune responses by virus-encoded immunomodulators. In particular, poxviruses evolved to counteract the NF-κB pathway by encoding multiple inhibitors targeting various levels of NF-κB signaling. We identified a NF-κB inhibitor encoded by ORFV, ORFV073, that is unique to Parapoxvirus (PPV). In contrast to previously described poxviral NF-κB inhibitors, ORFV073 is a virion protein available immediately following virus entry. Consistent with this possibility, ORFV073 efficiently inhibited NF-κB signaling very early during infection. Results also showed that this inhibition is important for ORFV pathogenesis in the natural host. Regulation of NF-κB signaling by virion proteins early in infection may be more prevalent among poxviruses and of greater biological significance than currently appreciated.

## Introduction

Orf virus (ORFV), the prototype member of the genus Parapoxvirus (PPV) of the *Poxviridae*, is the etiologic agent of contagious pustular dermatitis or orf, a ubiquitous disease of sheep and goats [1]. Orf is characterized by inflammatory, often proliferative lesions affecting the skin and the oral mucosa [2]. Lesions evolve through the stages of erythema, pustules and scabs, and are usually restricted to areas surrounding the virus entry sites [1,2]. If not complicated by secondary infections, orf lesions usually resolve in 6 to 8 weeks [3].

ORFV is highly epitheliotropic, and only keratinocytes or their counterparts in the oral mucosa have been shown to support viral replication *in vivo* [4,5]. Keratinocytes provide the first physical barrier to invading pathogens, and function as immune sentinels initiating inflammation and promoting skin healing after injury [6]. Keratinocytes express different cytokine receptors, such as tumor necrosis factor (TNF) receptor 1 (TNFR1) and interleukin-1 receptor (IL-1R), and multiple pattern recognition receptors (PRRs) such as toll-like receptors (TLRs) for recognition of pathogen-associated molecular patterns (PAMPs) of bacterial or viral origin [7]. Additional PRRs, such as the cyclic GMP-AMP Synthase (cGAS), retinoic acid -inducible gene 1 (RIG-I)-like receptors and NOD-like receptors (NLRs) recognize viral nucleic acid in the cytoplasm [8]. Engagement of these receptors initiates downstream pro-inflammatory signaling cascades [6,7], including the nuclear factor-kappa B (NF-κB) signaling pathway, which mediates innate immune responses and contributes to skin homeostasis [9,10].

NF-κB comprises multiple transcription factors (NF-κB-p65 [RelA], RelB, c-Rel, NF-κB-p50/p105 and NF-κB-p52/p100) that bind as homo- or heterodimers to specific DNA regulatory sequences to control expression of a wide range of cellular genes involved in innate immunity, inflammation, cell proliferation and differentiation, and apoptosis [11–13]. In unstimulated cells, NF-κB dimers are sequestered in the cytoplasm through binding to the inhibitor kappa-B alpha (IκBα) [13]. Most TLRs and IL-1 receptors transmit signals to the IκB kinase (IKK) complex via adaptor proteins interleukin receptor-associated kinase 1(IRAK1) and TNF Receptor Associated Factor 6 (TRAF6). However, TNFR1, TLR3 and TLR4 rely on Receptor-interacting protein kinase 1 (RIPK1) for activation of the IKK complex [14]. The IKK complex consists of the regulatory subunit IKKγ/NF-κB essential modulator (NEMO) and two kinases, IKKα and IKKβ [15]. In the canonical NF-κB pathway, various stimuli lead to phosphorylation of IκBα via IKKβ resulting in IκBα ubiquitination and subsequent proteasomal degradation [11,13]. Released p65/p50 dimers translocate to the nucleus where they bind κB-responsive DNA elements, recruit transcription co-regulators, and activate or repress gene expression [16]. Binding of NF-κB subunits to κB responsive elements and effective recruitment of transcriptional partners, however, are tightly regulated by posttranslational modifications of the NF-κB transcription complex and/or histones surrounding NF-κB target genes [16].

Given the central role played by NF-κB in regulating and integrating cellular processes such as inflammation and apoptosis it is not surprising that viruses have evolved strategies to counteract the NF-κB signaling pathway [17]. Poxviruses, in particular, are known to encode many NF-κB inhibitors, with selected viruses encoding multiple inhibitory functions [18,19]. Notably, poxviral NF-κB inhibitors target mainly cytoplasmic events leading to NF-κB activation [18,19]. For example, vaccinia virus (VACV) encodes at least ten cytoplasmic NF-κB inhibitors that target events leading to activation of the IKK complex (A52R, A46R, B14, C4, N1L, K7 and M2L), degradation of IκBα (A49, K1L), or activation of the protein kinase RNA (PKR)-double-stranded RNA (dsRNA) signaling pathway (E3L) [20–28]. Similarly, ectromelia virus, the causative agent of mousepox, encodes four F-box and ankyrin domain-containing proteins (EVM002, EVM005, EVM154 and EVM165) and a BTB/Kelch protein (EVM150) that prevent IκBα degradation and NF-κB-p65 nuclear translocation, respectively, by modulating ubiquitin ligase activity [29–31]. Recently, a molluscum contagiosum virus (MCV)-encoded protein MC132 was shown to inhibit NF-κB activation by interacting with- and targeting NF-κB-p65 for proteasomal degradation [32].

Several poxviral proteins specifically target the IKK complex, a bottleneck for most NF-κB activating signals, including those involved in nucleic acid sensing and response to infection [19]. Two VACV proteins prevent phosphorylation and subsequent activation of IKK complex. VACV B14 directly interacts and inhibits the activity of IKKβ, while VACV N1L interacts with multiple subunits of the IKK complex [21,23]. MCV FLICE-like proteins (vFLIPs) MC159 and MC160 also target the IKK complex, with MC159 interacting with NEMO and preventing activation of IKKβ and MC160 inducing cytoplasmic degradation of IKKα [33,34].

In general, multiple NF-κB inhibitors encoded by a given poxvirus function at different levels of the NF-κB signaling pathway; however, viruses encoding inhibitors acting at the same level have been described [19]. While targeting multiple branches of the NF-κB pathway, poxviral inhibitors are not completely redundant *in vivo* as viruses harboring single gene deletions affecting NF-κB inhibitors have been shown to influence aspects of disease [35].

Notably, apart from VACV E3L (ORFV020), homologues of the known poxviral inhibitors of NF-κB are absent in parapoxviruses, suggesting that these viruses have evolved novel proteins to counteract the NF-κB signaling pathway. Recently, we have described three NF-κB inhibitors encoded by ORFV, ORFV024, ORFV002, and ORFV121 [36–38]. ORFV024 was shown to inhibit phosphorylation of IKK kinases, thus preventing activation of IKK complex. ORFV121, a virulence determinant, was shown to bind to- and inhibit phosphorylation and nuclear translocation of NF-κB-p65. And, ORFV002 was shown to inhibit nuclear phosphorylation of NF-κB-p65 by interfering with NF-κB-p65 and mitogen- and stress activated kinase-1 (MSK1) interaction [37,39].

Here, we show that ORFV073, a virion protein unique to parapoxviruses, is an inhibitor of NF-κB signaling that prevents activation of the IKK complex and subsequent nuclear translocation of NF-κB-p65 at early times post-infection. Notably, ORFV073 represents the first poxviral virion-associated NF-κB inhibitor described, highlighting the significance of viral inhibition of NF-κB signaling very early in infection.

## Materials and methods

### Cells and viruses

Primary ovine fetal turbinate cells (OFTu) were kindly provided by Howard D. Lehmkuhl (USDA) and were maintained at 37°C with 5% CO_2_ in minimal essential medium (MEM) supplemented with 10% fetal bovine serum (FBS), 2 mM L-glutamine, 50 μg/ml gentamicin, 100 IU/ml penicillin, and 100 μg/ml streptomycin. HeLa cells (American Type Culture Collection) stably expressing green fluorescent protein (GFP) (GFP/HeLa) or ORFV073-GFP (073GFP/HeLa) fusion protein were maintained in Dulbecco's modified essential medium (DMEM) supplemented as above with the addition of neomycin (G418; 500 μg/ml; Gibco). ORFV strain OV-IA82 [40] was used to construct an OV-IA82 ORFV073 gene deletion virus (OV-IA82Δ073) and for experiments involving wild-type virus infection. OV-IA82Δ073 was used as parental virus to construct a flag tagged ORFV073 revertant virus (OV-IA82RV073^Flag^).

### Plasmids

To construct ORFV073-His expression plasmid, the ORFV073 coding sequence was PCR-amplified from the OV-IA82 genome with primers 073His-Fw(*HindIII*)-5’ TAATAAAT*AAGCTT*AAAATGGCGGGACGCGCGCGTTTTTC-3’and 073His-Rv(*EcoRI*)-5’-GACTTCGC*GAATTC*GGGGCAGTAGTTACAAAAACGTTT-3’ and cloned into the vector pcDNA/V5-His (Thermo Fisher Scientific, Waltham, MA). Similarly, to construct ORFV073-GFP (ORFV073-GFP) expression plasmid, the ORFV073 coding sequence was PCR-amplified from the OV-IA82 genome with primers 073GFP-Fw(*XhoI*)-5’-AGAAT*CTCGAG*ATGGCGGGACGCGCGCGTTTTTC-3’ and 073GFP-Rv(*BamHI*)-5’-AGCACT*GGATCC*GGGGCAGTAGTTACAAAAAC-3’ and cloned into the vector pEGFP-N1 (Clontech, Mountain View, CA). DNA sequencing of plasmids confirmed fidelity of constructs.

pcDNA3.1+IBKG/C-(K)-DYK and pcDNA3.1+TRAF6/C-(K)-DYK expression plasmids for NF-κB essential modifier (NEMO) and TNF receptor associated factor 6 (TRAF6), respectively were purchased from Genscript (Piscataway, NJ). Plasmid pCMV-RIPK1 for receptor-interacting protein kinase 1 (RIPK1) was kindly provided by Dr. Lin-Feng Chen (Department of Biochemistry, University of Illinois at Urbana-Champaign).

### Construction and characterization of ORFV073 deletion mutant virus OV-IA82Δ073 and ORFV073 revertant virus OV-IA82RV073^Flag^

To generate OV-IA82Δ073, a recombination cassette containing ORFV073 left (LF; 526 bp) and right (RF; 526 bp) flanking regions, and GFP gene driven by vaccinia virus VV7.5 promoter was synthesized and cloned in vector pUC57 (pUC57-073LF-GFP-073RF) (Genscript, Piscataway, NJ). Similarly, to generate OV-IA82RV073^Flag^, ORFV073 left (LF; 586 bp) and right (RF; 586 bp) flanking regions with ORFV073 coding sequence in frame with 3xflag sequence, and red fluorescent protein (RFP) sequences driven by VV7.5 promoter was synthesized and cloned into vector pUC57 (pUC57-073LF-0733xflag-RFP-073RF) (Genscript, Piscataway, NJ). DNA sequencing of constructs confirmed sequence integrity and identity.

To obtain OV-IA82Δ073, OFTu cells were infected with OV-IA82 and transfected with recombination vector pUC57-073LF-GFP-073RF. Similarly, to obtain OV-IA82RV073^Flag^, cells were infected with OV-IA82Δ073 and transfected with recombination vector pUC57-073LF-0733xflag-RFP-073RF. Fluorescent plaques indicative of recombinant virus replication were selected and subjected to virus purification by limiting dilution and plaque assays as previously described [37]. Integrity and fidelity of sequences in recombinant viruses were confirmed by PCR and DNA sequencing.

### Virus purification and characterization

To obtain semi-purified ORFV for infection experiments, OFTu cells cultured in five T175 were infected with OV-IA82, OV-IA82Δ073 or OV-IA82RV073^Flag^ (multiplicity of infection, MOI = 0.1) and harvested at 90–95% cytopathic effect (CPE). Cultures were freeze and thawed three times, spun down (1500 rpm, 5 min) to remove cellular debris, and then ultracentrifuged (25000 rpm, 1 h) to pellet virus. Virions were resuspended in MEM, and viral titers were determined by the Spearman and Karber’s method and expressed as tissue culture infectious dose 50 (TCID50)/ml.

For virion protein studies, OV-IA82Δ073 and OV-IA82RV073^Flag^ were purified by double sucrose gradients with modifications [41]. OFTu cells (10 T175 flasks) were infected with OV-IA82Δ073 or OV-IA82RV073^Flag^ (MOI = 0.1), harvested at advanced CPE, and centrifuged to obtain supernatant and cell pellet fractions. Supernatants were ultracentrifuged to pellet extracellular enveloped virus (EEV) as described above and cell pellets were freeze and thawed three times to release intracellular mature virus (IMV) and centrifuged to remove cellular debris. Both EEV and IMV preparations were centrifuged through a sucrose cushion followed by double sucrose gradient purification. EEV and IMV-containing bands were collected and resuspended in 250 μl 10 mM Tris Hcl. Whole cell lysates (10 μg) from mock and OV-IA82RV073^Flag^ infected cells (MOI = 10) (24 h p.i) and purified OV-IA82Δ073 and OV-IA82RV073^Flag^ EEV and IMV virion proteins (10 μg) were resolved by SDS-PAGE, blotted to nitrocellulose membrane and probed with primary antibody against flag (Catalog no. A00187-200; Genscript) or ORFV086 structural protein [42]. Blots were developed with HRP-conjugated goat anti mouse secondary antibody (sc-2031; Santa Cruz) and chemiluminescent reagent (Super Signal West Femto, Thermo Fischer).

### Establishment of ORFV073 expressing stable cell lines

A retroviral expression system (pLNCX2; Clontech) was used to construct HeLa cells constitutively expressing GFP (GFP/HeLa) or ORFV073-GFP (ORFV073GFP/HeLa) fusion protein. GFP or ORFV073-GFP DNA sequences were cloned into plasmid pLNCX2 and transfected into the packaging cell line GP2-293 using Lipofectamine 2000. After 48 h, supernatants containing GFP or ORFV073-GFP-encoding recombinant retrovirus particles were harvested and used to infect HeLa cells. Selection, amplification and maintenance of the individual clones were performed in the presence of G418 (500 μg/ml; Gibco). Expression of control GFP or ORFV073-GFP was monitored by fluorescence microscopy and Western blot using antibody against GFP (sc-9996; Santa Cruz Biotechnology).

### ORFV073 sequence analysis

Analysis of ORFV073 sequence and prediction of subcellular localization was performed with PSORT II (https://psort.hgc.jp/form2.html), NoLS (http://www.compbio.dundee.ac.uk/www-nod/) and NucPred (http://www.sbc.su.se/~maccallr/nucpred/). Alignment of PPV ORFV073 amino acid sequences was performed using CLUSTAL Omega (EMBL-EBI). Virus strains and GenBank accession numbers used for the alignment are as follows: BPSV strain BV-AR02, NC 005337.1; PPV red deer (PPV-RD) strain HL953, NC 025963.1; PCPV strain F00.120R, GQ329669.1; ORFV strains D1701, HM133903.1; NA1/11, KF234407.1; NZ2, DQ184476.1; IA82, AY386263.1; B029, KF837136.1; OV-SA00, NC 005336.1; GO, KP010354.1; NP, KP010355.1; SJ1, KP010356.1; YX, KP010353.1.

### ORFV073 protein expression and subcellular localization

To assess ORFV073 protein expression, OFTu cells were mock infected or infected with OV-IA82RV073^Flag^ (MOI = 10) for 2, 4, 6, 8, 10, 12 or 24 h post infection (h p.i.). Whole cell protein extracts (50 μg) were resolved by SDS-PAGE, blotted to nitrocellulose membranes and probed with primary antibody against flag and glyceraldehyde-3-phosphate dehydrogenase (GAPDH) (sc-25778; Santa Cruz). Blots were developed with appropriate HRP-conjugated secondary antibodies (sc-2031 and sc-2004; Santa Cruz) and chemiluminescent reagents.

The transcription kinetics of *ORFV073* during ORFV infection in OFTu cells was examined by RT-PCR following procedures previously described [36]. Transcription of *ORFV073*, *ORFV085* (late gene control) and *ORFV127* (early gene control) was assessed by PCR using the primers 073GFP-Fw(*XhoI*) and 073GFP-Rv(*BamHI*) (described above), 085LintFw-5’-ACGCCTAGCAGCAGGTACA-3’ and 085LintRv-5’-GCTACGTGACGGTGATCAAG-3’, and 127EintFw-5’-CTCCTCGACGACTTCAAAGG-3’ and 127EintRv-5’-TATGTCGAACTCGCTCATGG-3’ respectively.

To determine the subcellular localization of ORFV073 and structural protein ORFV086, OFTu cells cultured in chamber slides (ibidi, Martinsried, Germany) were mock infected or infected with OV-IA82RV073^Flag^ and OV-IA82, respectively (MOI = 10). At 30 min and 1, 2, 6, 8, 12, 16 and 24 h p.i., cells were fixed with 4% formaldehyde for 20 min, permeabilized with 0.2% Triton-X for 10 min, blocked with 1% bovine serum albumin for 1 h and then incubated with primary mouse monoclonal antibody against flag (no. A00187-200; Genscript) or ORFV086 [42] overnight at 4°C. Cells were then incubated with Alexa Fluor 488-labeled secondary goat anti mouse antibody (no. A-11001; Thermo Fisher Scientific) for 1 h, counterstained with DAPI for 10 min, and examined by confocal microscopy (A1, Nikon).

To examine the possibility of localization of ORFV073 in endosomes, co-localization of ORFV073 with endosomal marker (Caveolin-1) was performed. OFTu cells mock infected or infected with OV-IA82RV073^Flag^ (MOI = 10) were fixed at 16 and 24 h p.i, permeabilized and blocked as describe above. Cells were sequentially incubated with primary mouse monoclonal antibody against flag (no. A00187-200; Genscript) and rabbit polyclonal antibody against Caveolin-1 (no. sc-894, Santa Cruz), and Alexa Fluor 488-labeled secondary goat anti mouse antibody (no. A-11001; Thermo Fisher Scientific) and Alexa Fluor 647-labeled secondary goat anti rabbit antibody (no. A-21244; Thermo Fisher Scientific). Cells were then counterstained with DAPI and examined by confocal microscopy (A1, Nikon)

To examine co-localization of ORFV073 with ORFV086, OFTu cells mock infected or infected with OV-IA82RV073^Flag^ (MOI = 10) were fixed at 16 and 24 h p.i, permeabilized and blocked as describe above. Cells were sequentially incubated with primary rabbit monoclonal antibody against flag (no. 14793, Cell Signaling) and mouse monoclonal antibody against ORFV086, and Alexa Fluor 488-labeled secondary goat anti rabbit antibody (no. A-11008; Thermo Fisher Scientific) and Alexa Fluor 647-labeled secondary goat anti mouse antibody (no. A-21236; Thermo Fisher Scientific). Cells were then counterstained with DAPI and examined by confocal microscopy (A1, Nikon).

### Growth curves

The replication characteristics of OV-IA82Δ073 was assessed in OFTu cells. Cells cultured in 6-well plates were infected with OV-IA82 or OV-IA82Δ073 using MOI 0.1 (multi-step growth curve) or 10 (single-step growth curve) and harvested at 6, 12, 24, 36, 48, 72 and/or 96 h p.i. Virus titers at each time point were determined as described above. To compare the cytopathic effect (CPE) induced by OV-IA82 and OV-IA82Δ073, OFTu cells were infected with OV-IA82 or OV-IA82Δ073 (MOI = 10) and evaluated under an inverted light microscope at 48 h p.i. (Leica DMI 4000B; 20X).

### Real-time PCR analysis

To assess the effect of ORFV073 on NF-κB regulated gene transcription, OFTu cells were mock infected or infected with OV-IA82, OV-IA82Δ073 or OV-IA82RV073^Flag^ (MOI = 10) and harvested at 1 and 2 h p.i. in the presence of Trizol reagent (Thermo Fisher, Waltham, MA), and RNA samples were processed and reverse transcribed as previously described [36]. The expression of interleukin-8 (IL-8), prostaglandin endoperoxide synthase 2 (PTGS2), C-C chemokine ligand 20 (CCL20) and NF-κB inhibitor alpha (NF-κBIA) genes was assessed using Custom Plus TaqMan Gene Expression Assays (Applied Biosystems) based on ovine gene sequences in GenBank. Real-time PCR and data analysis were performed as previously described [36]. Statistical analysis of the data was performed by using Student’s t test.

### NF-κB-p65 nuclear translocation assay

To investigate the effect of ORFV073 on nuclear translocation of NF-κB-p65 following ORFV infection, OFTu cells were mock infected or infected (MOI = 10) with OV-IA82, OV-IA82Δ073 or OV-IA82RV073^Flag^. Cells were fixed at 30 min and 1, 2, 4, 6, 8, 12 and 24 h p.i. as described above, sequentially incubated with antibody against NF-κB-p65 (no. 8242; Cell Signaling) and with Alexa Fluor 488-labeled goat anti rabbit antibody, counterstained with DAPI, and examined by confocal microscopy. Cells (n = approximately 300 per sample) from randomly selected fields were scored for nuclear NF-κB-p65 and results depicted as the mean percentage of cells expressing nuclear NF-κB-p65 for each time point. Statistical analysis of data was performed by using Student’s t test.

To examine the effect of ORFV073 expression on TNFα-induced nuclear translocation of NF-κB-p65, HeLa cells stably expressing GFP (GFP/HeLa) or ORFV073-GFP fusion protein (073GFP/HeLa) were treated with 20 ng/ml of TNFα (Cell Signaling, Danvers, MA). Cells were fixed at 30 min and 1 h post-treatment, sequentially incubated with primary antibody against NF-κB-p65, and Alexa Fluor 594-labeled goat anti rabbit secondary antibody (no. A-11037, Thermo Fisher Scientific), counterstained with DAPI, and examined by confocal microscopy. Cells (n = approximately 200 per sample) from randomly selected fields were scored for nuclear NF-κB-p65 and results depicted as the mean percentage of GFP/073GFP expressing cells containing nuclear NF-κB-p65 for each time point. Statistical analysis of data was performed by using Student’s t test.

To evaluate the effect of protein synthesis inhibitor cycloheximide (CHX) on nuclear translocation of NF-κB-p65 during ORFV infection, OFTu cells mock treated or treated with CHX (50 μg/ml) (Sigma-Aldrich, St. Louis, MO) for 30 min were mock infected or infected with OV-IA82, OV-IA82Δ073 or OV-IA82RV073^Flag^ (MOI = 10) in absence or presence of CHX (50 μg/ml) for 1 h. Nuclear translocation assays and data analysis were performed as described above. As a control for CHX activity, OFTu cells mock treated or treated with CHX (50 μg/ml) for 30 min were mock infected or infected with OV-IA82RV073^Flag^ and harvested at 30 min and 1 h p.i. Whole cell protein extracts (50 μg) were resolved by SDS-PAGE, and transferred to nitrocellulose membranes and probed with antibody against p53 (sc-6243; Santa Cruz) and actin (sc-8432; Santa Cruz) as described above.

### Effect of ORFV073 on NF-κB-p65 signaling pathway

HeLa cells stably expressing GFP (GFP/HeLa) or ORFV073-GFP (073GFP/HeLa) were treated with TNFα (20 ng/ml) and harvested at 5, 10 and 15 min post treatment. OFTu cells mock infected or infected with OV-IA82, OV-IA82Δ073 or OV-IA82RV073^Flag^ (MOI = 10) were harvested at 30 min and 1 h p.i. Whole cell protein extracts (50 μg) were resolved by SDS-PAGE, blotted to nitrocellulose membranes and probed with antibody against phospho-IKKα/β (Ser176/180) (no. 2697; Cell Signaling), phospho-IκBα (Ser32/36) (no. 9246; Cell Signaling), phospho-NF-κB-p65 (Ser536) (no. 3033; Cell Signaling), IKKα/β (sc-7607; Santa Cruz), IκBα (sc-371; Santa Cruz), NF-κB-p65 (sc-7151; Santa Cruz), GAPDH or GFP (sc-9996; Santa Cruz). Blots were processed as described above. Densitometric analysis of the blots was performed with ImageJ software version 1.6.0 (National Institutes of Health, Bethesda, MD). Statistical analysis of densitometry data was performed by using the Student’s t test.

To investigate the kinetics of NF-κB activation following ORFV infection, OFTu cells were infected with OV-IA82 or OV-IA82Δ073 (MOI = 10) and harvested at 30 min, 1 h, 2 h, 4 h, 6 h, 8 h and 12 h p.i. Whole cell protein extracts (50 μg) were resolved by SDS-PAGE, blotted to nitrocellulose membranes, probed with phospho-NF-κB-p65, NF-κB-p65 and GAPDH antibodies, and developed as described above.

To assess the effect of ORFV073 on NEMO levels, OFTu cells, mock infected or infected with OV-IA82 or OV-IA82Δ073 (MOI = 10) were harvested at 30 min, 45 min, 1 h, 1 h 30 min and 2 h p.i., and cytoplasmic protein extracts were prepared using NE-PER Nuclear and Cytoplasmic Extraction Reagents following manufacturer’s protocol (Thermo Fisher, Waltham, MA). Extracts (50 μg) were resolved by SDS-PAGE, blotted to nitrocellulose membranes, probed with NEMO (sc-8330, Santa Cruz) and GAPDH antibodies, and developed as described above. Densitometric and statistical analysis of the blots was performed as described above.

### Co-immunoprecipitation assays

To investigate the potential interaction of ORFV073 with cellular proteins NEMO, RIPK1 and TRAF6, OFTu cells co-transfected with 1 μg of pcDNA/V5-His (control plasmid) or pcDNA/V5-073His (ORFV073-His) together with either pcDNA3.1-NEMO, pCMV-RIPK1 or pcDNA3.1-TRAF6 were harvested 24 h post transfection and nuclear extracts were prepared using NE-PER Nuclear and Cytoplasmic Extraction Reagents (Thermo Fisher, Waltham, MA). Co-immunoprecipiation was performed using Nuclear Complex Co-IP Kit (Active Motif, Carslbad, CA) following manufacturer’s protocols. Nuclear protein extracts were co-immunoprecipitated with antibodies against His (no. A00186; Genscript), NEMO (sc-8330, Santa Cruz), RIPK1 (no. 3493, Cell Signaling) or TRAF6 (sc-7221, Santa Cruz) overnight at 4°C, and then incubated with 50 μl of pre-washed protein G agarose beads (no. 16–266; Millipore) at 4°C for 2 h. Beads were washed four times with high stringency buffer and eluted proteins (2x Laemelli buffer) resolved by SDS-PAGE, blotted to nitrocellulose membranes, probed with antibodies against His, NEMO, RIPK1 or TRAF6 and developed as described above. Light chain specific secondary antibody against Rabbit IgG (no. ab99697; Abcam) was used for NEMO blots.

### Animal inoculations

To evaluate the effect of ORFV073 on ORFV virulence in the natural host, five-month-old lambs were randomly allocated to three experimental groups, OV-IA82Δ073 (n = 4), OV-IA82RV073^Flag^ (n = 4) and mock (n = 3). Following anesthesia, the mucocutaneous junction of the lip near the right labial commissure and the inner sides of hind limbs were scarified along 2 cm and 5 cm-long lines, respectively, and virus inoculum (0.5 ml) containing 10^7.5^ TCID50/ml was applied topically to each inoculation site using cotton swabs. The scarified areas of the lips were monitored for 21 days for the presence of characteristic orf lesions. Criteria assessed were extent of erythema, papules, pustules, and attached scab. Each criterion was scored according to the width of the lesion along the line of scarification: 1, lesion < 0.5 cm across; 2, lesion 0.5 cm-1 cm across; 3, lesion > 1 cm across, and the total daily score for each lamb was the sum of scores of the four lesion types. Skin biopsy specimens were collected at days 2, 5, 8, 12 and 21 p.i., fixed in 10% buffered formalin, embedded in paraffin, sectioned, and stained with hematoxylin and eosin using standard methods.

### Ethics statement

All animal procedures were approved by University of Nebraska-Lincoln Institutional Animal Care and Use Committee (IACUC; protocol 1318) and were performed in accordance with the Guide for the Care and Use of Agricultural Animals in Agricultural Research and Teaching.

## Results

### ORFV073 is a conserved PPV protein that is expressed late during virus infection and localizes in the nucleus

**Fig 1:**
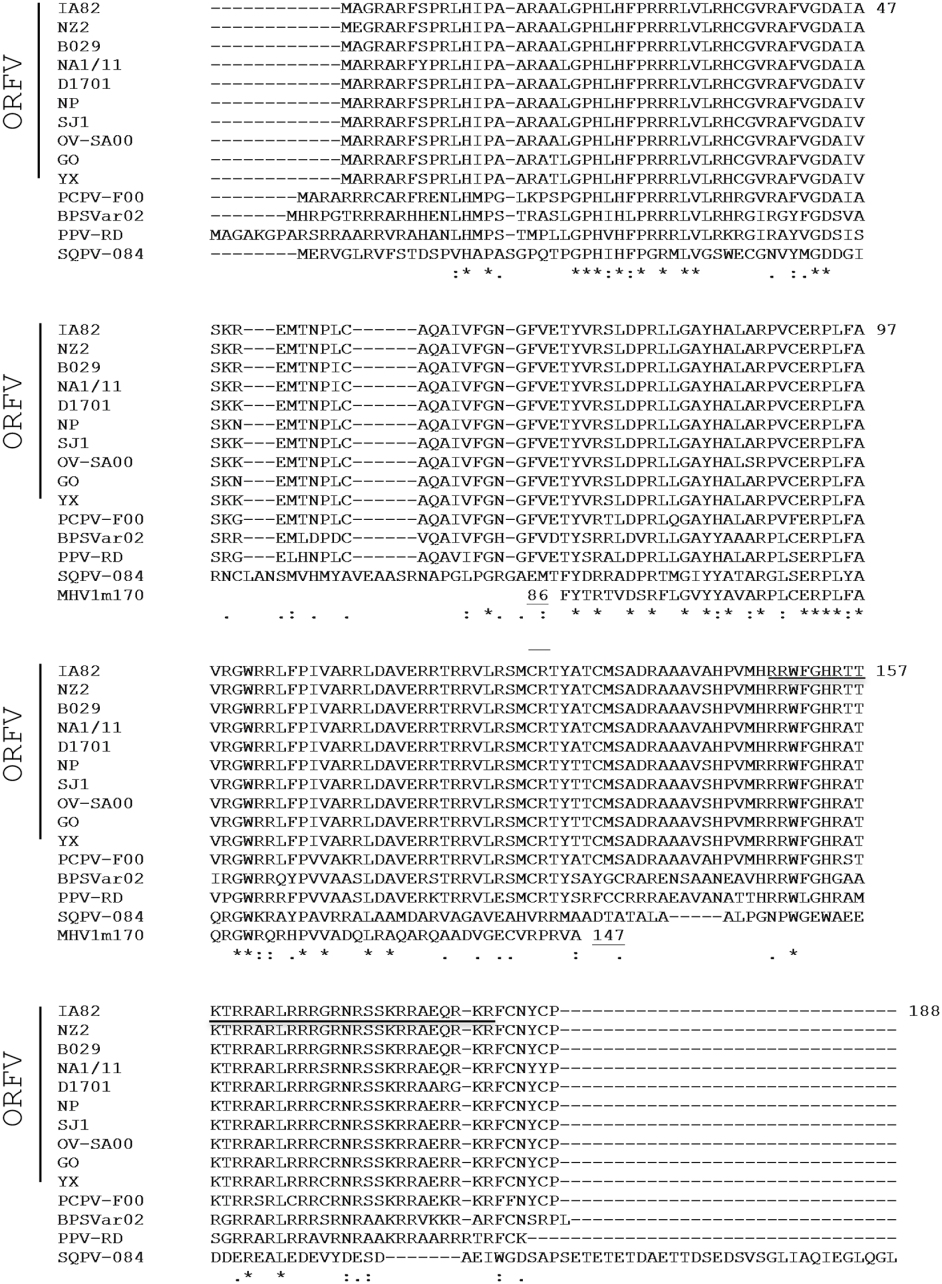
Clustal W alignment of amino acid sequences corresponding to parapoxvirus ORFV073 homologs, squirrellpox virus (SQPV)-084 protein, and murid betaherpesvirus m170 protein. The ORFV strain IA82 sequence is at the top of the alignment, with amino acid positions indicated by numbers on the right. The predicted nuclear localization signal is shown underlined. Sequences listed below strain IA82 are arranged in order of decreasing % amino acid identity relative to strain IA82. For clarity, only the murid betaherpesvirus m170 domain with homology to parapoxvirus ORFV073 proteins is shown (m170 positions 86–147). Asterisks [*], colons [:], and periods [.] below the alignment indicate fully, strongly, and weakly conserved, residues, respectively. Viruses, strains, and GenBank accession numbers (in parentheses) used for the alignment are as follows: ORFV strains OV-IA82 (AY386263.1), NZ2 (DQ184476.1), B029 (KF837136.1), NA1/11 (KF234407.1), D1701 (HM133903.1), NP (KP010355.1), SJ1 (KP010356.1), OV-SA00 (NC 005336.1), GO (KP010354.1), and YX (KP010353.1); Pseudocowpox virus strain F00.120R [PCPV-F00] (GQ329669.1); Bovine papular stomatitis virus strain BV-AR02 [BPSVar02] (NC 005337.1); Parapoxvirus of the red deer in New Zealand strain HL953 [PPV-RD] (NC 025963.1); Squirrellpox virus strain Red squirrel UK [SQPV-084] (YP_008658509); Murid betaherpesvirus 1 strain N1 [MHV1m170] (CCE57166).

*ORFV073* encodes for an arginine-rich 188-amino acid, basic protein with predicted molecular weight of 21.9 kDa. ORFV073 is highly conserved among ORFV isolates exhibiting 95%-99% amino acid identity (aa id), and less similar to orthologs in pseudocowpox virus (PCPV, 89% aa id), parapoxvirus of the Red Deer (PPV-RD, 70% aa id), and bovine papular stomatitis virus (BPSV, 63% aa id). Notably, PCPV contains two PPV073 paralogs arranged back to back in the genome (PCPV073 and PCPV073.5; 45% aa id), which are likely the result of gene duplication followed by divergent evolution [43]. A divergent ORFV073 homolog (SQPV0840, 36% aa id) is found in squirrelpox virus, a member of a novel chordopoxvirus genus closely related to PPV. Interestingly, mouse betaherpesvirus 1 (i.e. murid cytomegalovirus, a virus that circulates in wild mice) encodes a protein of unknown function (m170) similar in size to PPV073 and with a region of approximately 50 residues sharing 56% aa id to PPV073 (OV-IA82 amino acid positions 71–122) (Fig 1). While PPV073 orthologs contain a predicted nuclear localization signal (NLS) at their carboxyl-termini (OV-IA82 amino acid positions 149–182; underlined in Fig 1), no NLS was predicted for SQPV0840 and m170.

**Fig 2:**
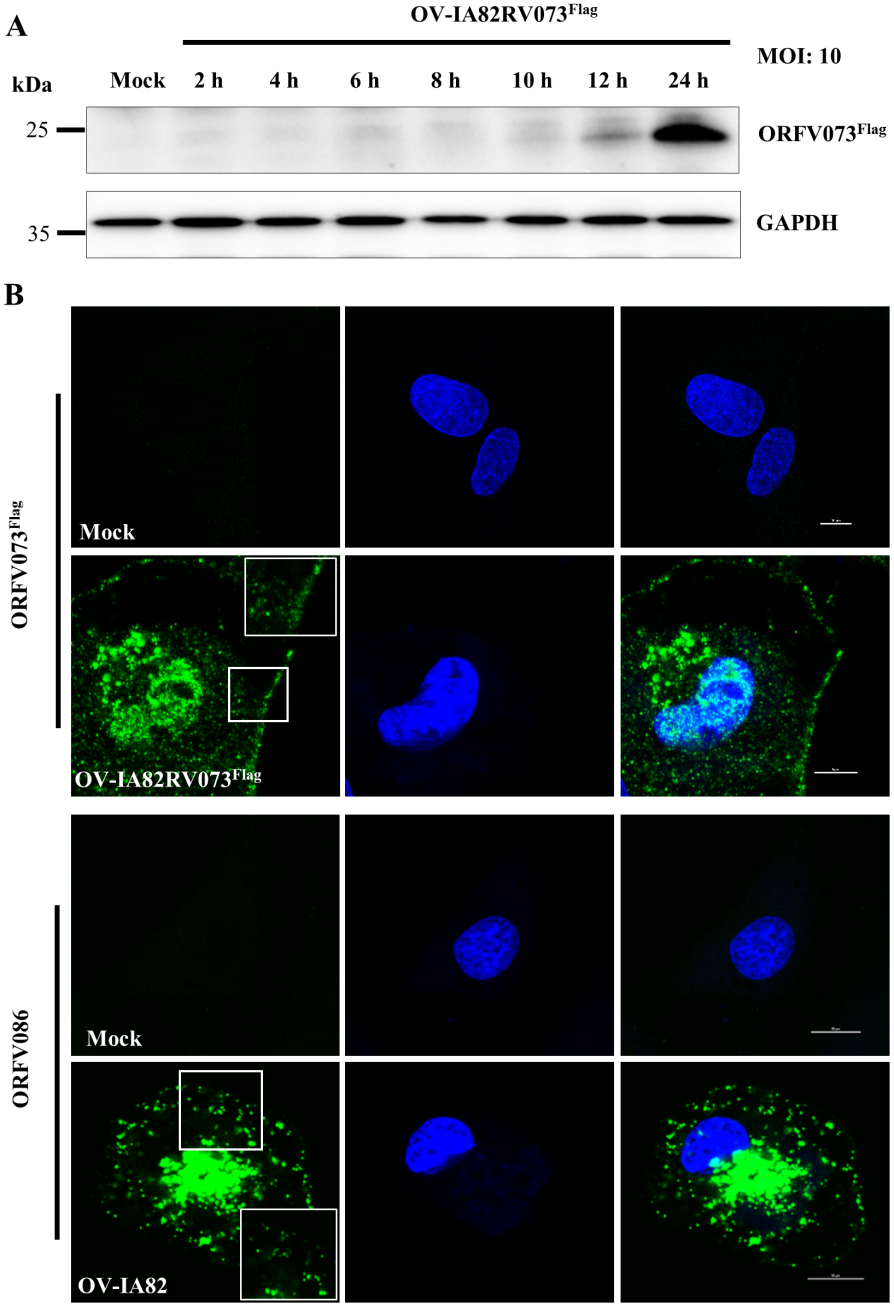
(A) ORFV073 protein expression kinetics. OFTu cells were mock infected or infected with ORFV073 revertant virus (OV-IA82RV073^Flag^) (MOI = 10) for 2, 4, 6, 8, 10, 12 and 24 h p.i. Whole cell protein extracts (50 μg) were resolved by SDS-PAGE, blotted to nitrocellulose membranes and probed with antibodies against flag and GAPDH (control). (B) Subcellular localization of ORFV073 (top panels) and structural protein ORFV086 (bottom panels) in OFTu cells mock infected or infected with revertant virus OV-IA82RV073^Flag^ or wild type virus OV-IA82, respectively (MOI = 10). Cells were fixed at 30 min and 1, 2, 6, 8, 12, 16 and 24 h p.i., incubated with anti-flag or anti-ORFV086 monoclonal antibodies, sequentially stained with Alexa Fluor 488-labeled secondary antibody and DAPI, and examined by confocal microscopy. Insets show magnified areas of the field. Shown are representative images from 24 h p.i. ORFV073 and ORFV086, green; DAPI, blue. Results are representative of two independent experiments.

The expression kinetics of ORFV073 was assessed by Western blot. ORFV073 was increasingly detected between 10 and 24 hours post-infection (h p.i.) (Fig 2A). Consistent with this observation, *ORFV073* transcription was detected only at late times during ORFV infection (6 to 24 h p.i.) (S1 Fig). ORFV073 transcripts were markedly decreased at 12 and 24 h p.i. in the presence of AraC, an inhibitor of DNA replication and of late poxviral gene transcription (S1 Fig). Together, these results indicate that ORFV073 belongs to the late class of poxviral genes.

To determine the subcellular localization of ORFV073, OFTu cells were mock infected or infected with OV-IA82RV073^Flag^ and examined by immunofluorescence at 30 min and 1, 2, 6, 8, 12, 16 and 24 h p.i as described in Material and Methods. Prior to the 16 h p.i sampling point, no convincing ORFV073 specific staining was observed in infected cells. ORFV073 was found predominantly in perinuclear regions and the nucleus of infected cells, and within small circular to ovoid structures (340.2±44.8nm) in proximity to the perinuclear region and the cell membrane at 16 and 24 h p.i. Similarly sized structures (387.4±51.6nm) were observed following staining for virion structural protein ORFV086 (Fig 2B). ORFV073 and ORFV086 co-localized in perinuclear regions and the smaller sized structures (S2A Fig). To rule out the possibility that the smaller ORFV073 stained structures were endosomes, co-localization studies of ORFV073 and endosomal marker (Caveolin-1) were performed. No co-staining was observed (S2B Fig). Results suggest that ORFV073, a late viral protein, may be a virion component.

### ORFV073 is nonessential for ORFV replication in primary OFTu cells

**Fig 3:**
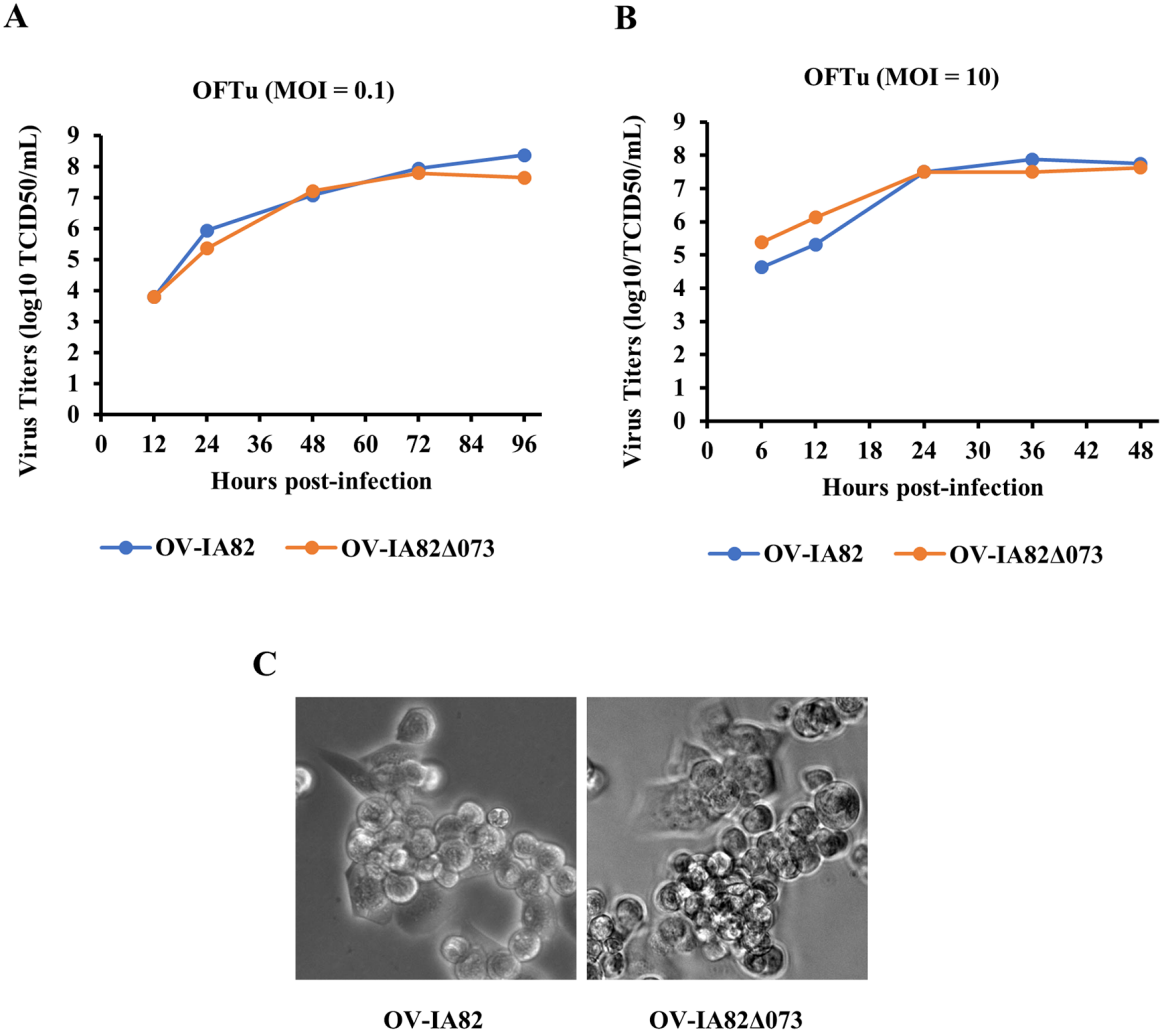
Replication characteristics of ORFV073-gene deletion virus. Primary OFTu cells were infected with wild-type OV-IA82 or deletion mutant OV-IA82Δ073 viruses and virus titers determined at various times p.i. as described in Material and Methods. (A) Multi-step growth curve, MOI 0.1; (B) single-step growth curve, MOI 10. (C) CPE of wild-type OV-IA82 or deletion mutant OV-IA82Δ073 virus infected OFTu cells at 48 h p.i (MOI = 10) (magnification, X20).

The replication kinetics of *ORFV073* gene deletion virus (OV-IA82Δ073) was compared with that of wild-type virus (OV-IA82) in primary ovine cells (OFTu). No differences in replication kinetics and viral yields were observed in multi-step or one-step growth curves between the two viruses (Fig 3A and 3B). Also, no differences in cytopathic effect, and plaque size and morphology were observed between the viruses (Fig 3C). These data indicate that ORFV073 is nonessential for ORFV replication in OFTu cells.

### ORFV073 inhibits NF-κB signaling early during infection

**Fig 4:**
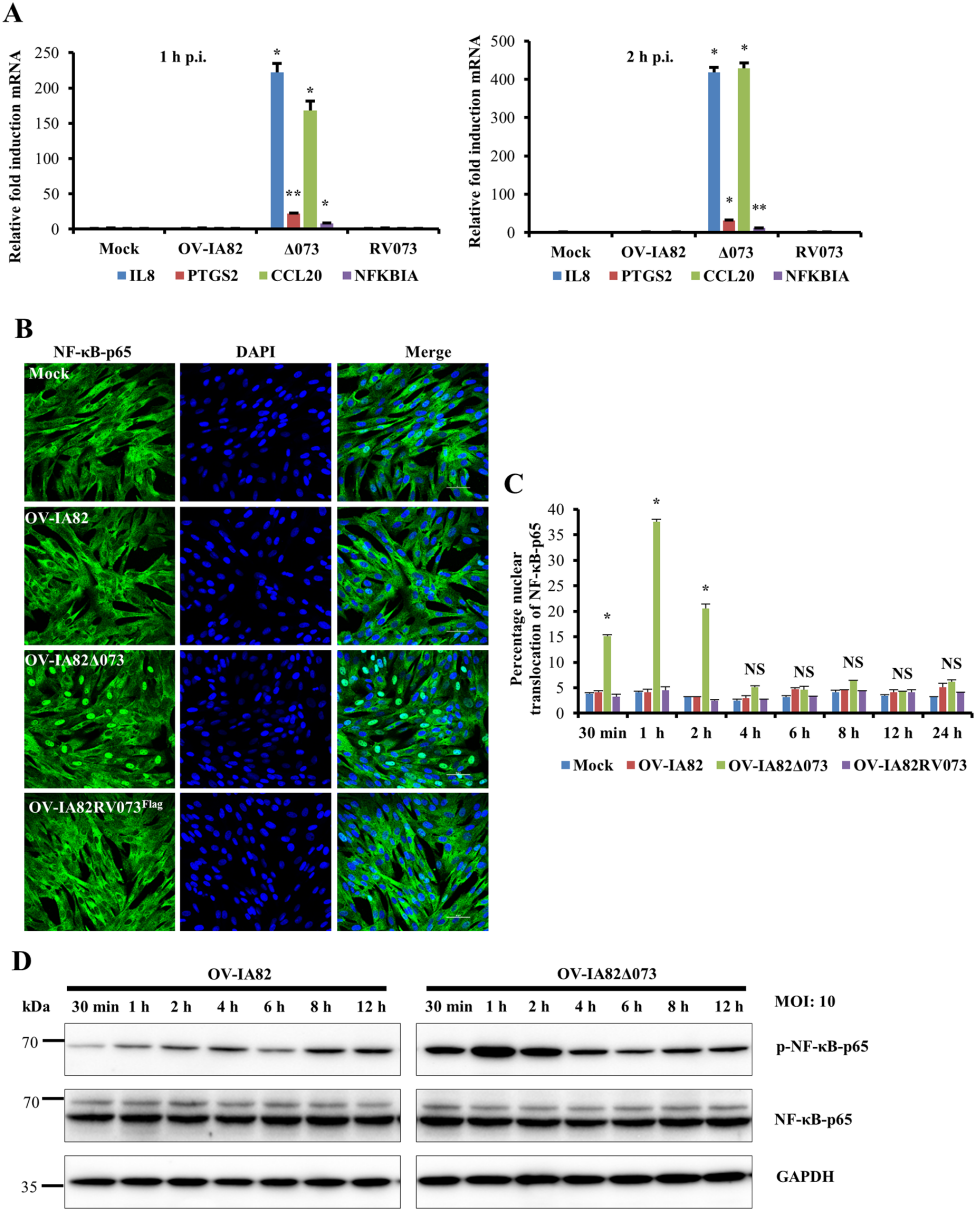
Effect of ORFV073 on NF-κB-p65 activation during ORFV infection. (A) OFTu cells were mock infected or infected with OV-IA82, OV-IA82Δ073 or OV-IA82RV073^Flag^ (MOI = 10) and transcription of selected NF-κB regulated genes was assessed by real-time PCR at 1 and 2 h p.i. (*, *P<0*.*05*, **, *P<0*.*01)*. Fold changes are relative to OV-IA82 treatment and data are mean mRNA levels from two independent experiments. (B) OFTu cells were mock infected or infected with OV-IA82, OV-IA82Δ073 or OV-IA82RV073^Flag^ (MOI = 10). Cells were fixed at 1 h p.i. and incubated with antibody against NF-κB-p65. Cells were then stained with Alexa Fluor 488-labeled secondary antibody and DAPI, and examined by confocal microscopy. Green, NF-κB-p65; blue, DAPI. (C) Cells (n = approximately 300) expressing nuclear NF-κB-p65 in each group were counted and results depicted as the mean percentage of cells containing nuclear NF-κB-p65 at the indicated time points post infection. Results are mean of two independent experiments (*, P<*0.05*). (D) OFTu cells were mock infected or infected with OV-IA82 or OV-IA82Δ073 (MOI = 10) and harvested at the indicated times p.i. Whole cell protein extracts (50 μg) were resolved by SDS-PAGE, blotted to nitrocellulose membranes and probed with antibodies against phospho or total NF-κB-p65, and GAPDH. Results in (B and D) are representative of two independent experiments.

On preliminary microarray analysis increased transcription of multiple NF-κB regulated genes MMP13 (8.5-fold), MMP1 (7.3-fold), CASP4 (3.4-fold) and IL-6 (2.5-fold) was observed in cells infected with OV-IA82Δ073 compared to cells infected with wild-type virus, suggesting that ORFV073 inhibits NF-κB function. To validate this observation, real-time PCR analysis of gene expression was conducted. To rule out any confounding effect from cytokines that potentially might be present in the virus inocula, viruses used in these studies were semi-purified as described in Materials and Methods. Increased transcription of NF-κB-regulated genes IL8 (222.1 and 418.6-fold), PTGS2 (22 and 31.2-fold), CCL20 (168.1 and 429.2-fold) and NFKBIA (8 and 12.2-fold) was observed in cells infected with OV-IA82Δ073 compared to wild type OV-IA82 at 1 and 2 h p.i., respectively (Fig 4A). To assess whether ORFV073 affects NF-κB-p65 nuclear translocation, OFTu cells were infected with OV-IA82, OV-IA82Δ073 or OV-IA82RV073^Flag^, or mock infected, and NF-κB-p65 localization was examined by immunofluorescence. Infection with OV-IA82Δ073 but not OV-IA82 or OV-IA82RV073^Flag^ led to rapid nuclear translocation of NF-κB-p65 as early as 30 minutes p.i. (Fig 4B and 4C). The effect was transient as levels of nuclear NF-κB-p65 returned to those in wild-type virus-infected cells between 2 and 4 h p.i. (Fig 4C, *P*<0.05). Consistent with the nuclear translocation kinetics, levels of phosphorylated NF-κB-p65 (Ser536), which accumulates in the cytoplasm prior to nuclear translocation, are increased at early times p.i. with OV-IA82Δ073 (Fig 4D). Together, data show that ORFV073 is a NF-κB inhibitor acting transiently very early in infection.

### Infection of cells with OV-IA82Δ073 leads to increased levels of NEMO and marked phosphorylation of IKKα/β, IκBα and NF-κB-p65 early in infection

**Fig 5:**
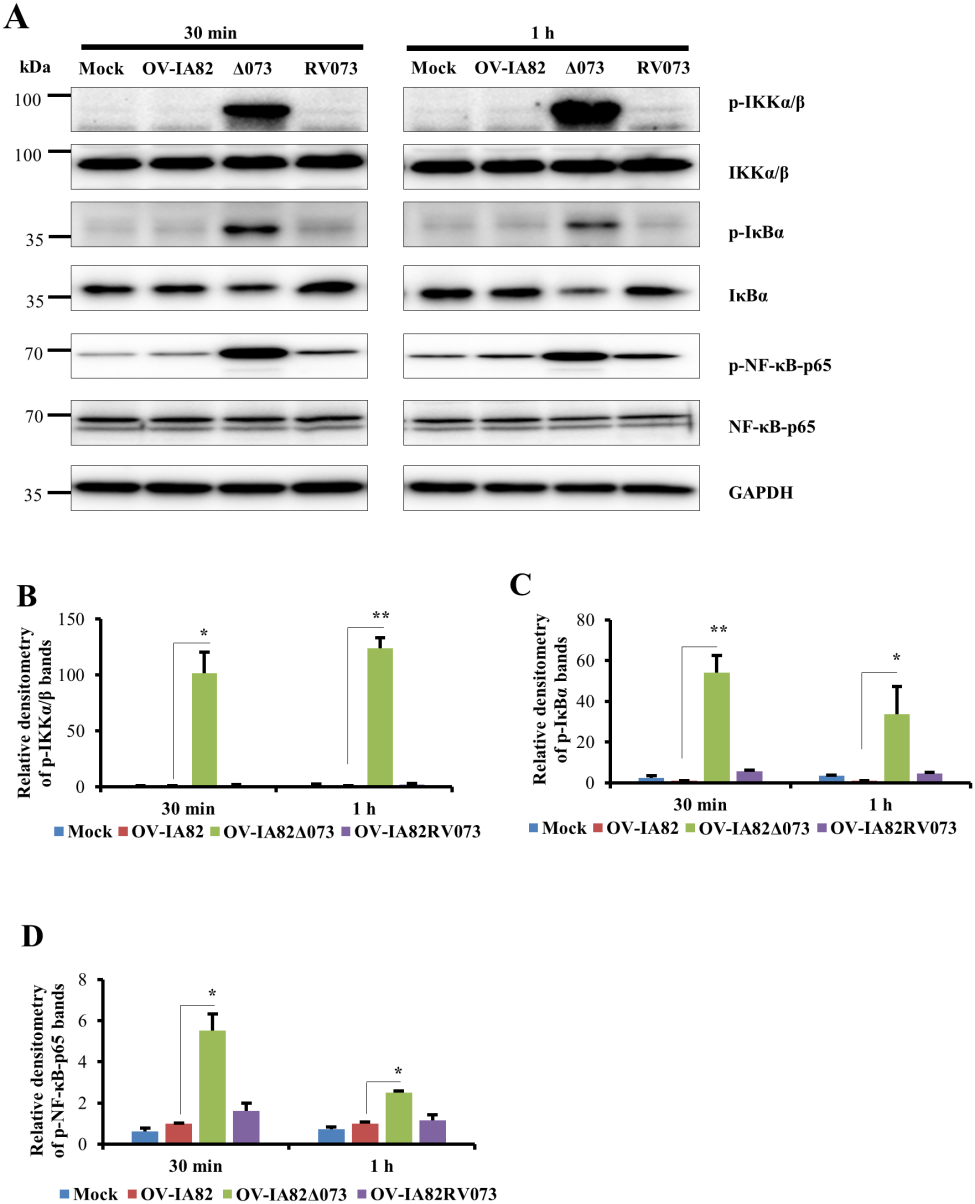
Effect of ORFV073 on phosphorylation of IKKα/β, IκBα and NF-κB-p65 during ORFV infection. (A) OFTu cells were mock infected or infected with OV-IA82, OV-IA82Δ073 or OV-IA82RV073^Flag^ (MOI = 10) and harvested at 30 min and 1 h p.i. Whole cell protein extracts (50 μg) were resolved by SDS-PAGE, blotted to nitrocellulose membranes and probed with antibodies against phospho and total IKKα/β, IκBα and NF-κB-p65, and GAPDH. (B-D) Densitometry analysis of p-IKKα/β, p-IκBα and p-NF-κB-p65, respectively; bands were normalized to the loading control GAPDH. Fold changes are shown relative to OV-IA82 and data are mean values of three independent experiments (*, *P<0*.*05* and **, *P<0*.*01*).

**Fig 6:**
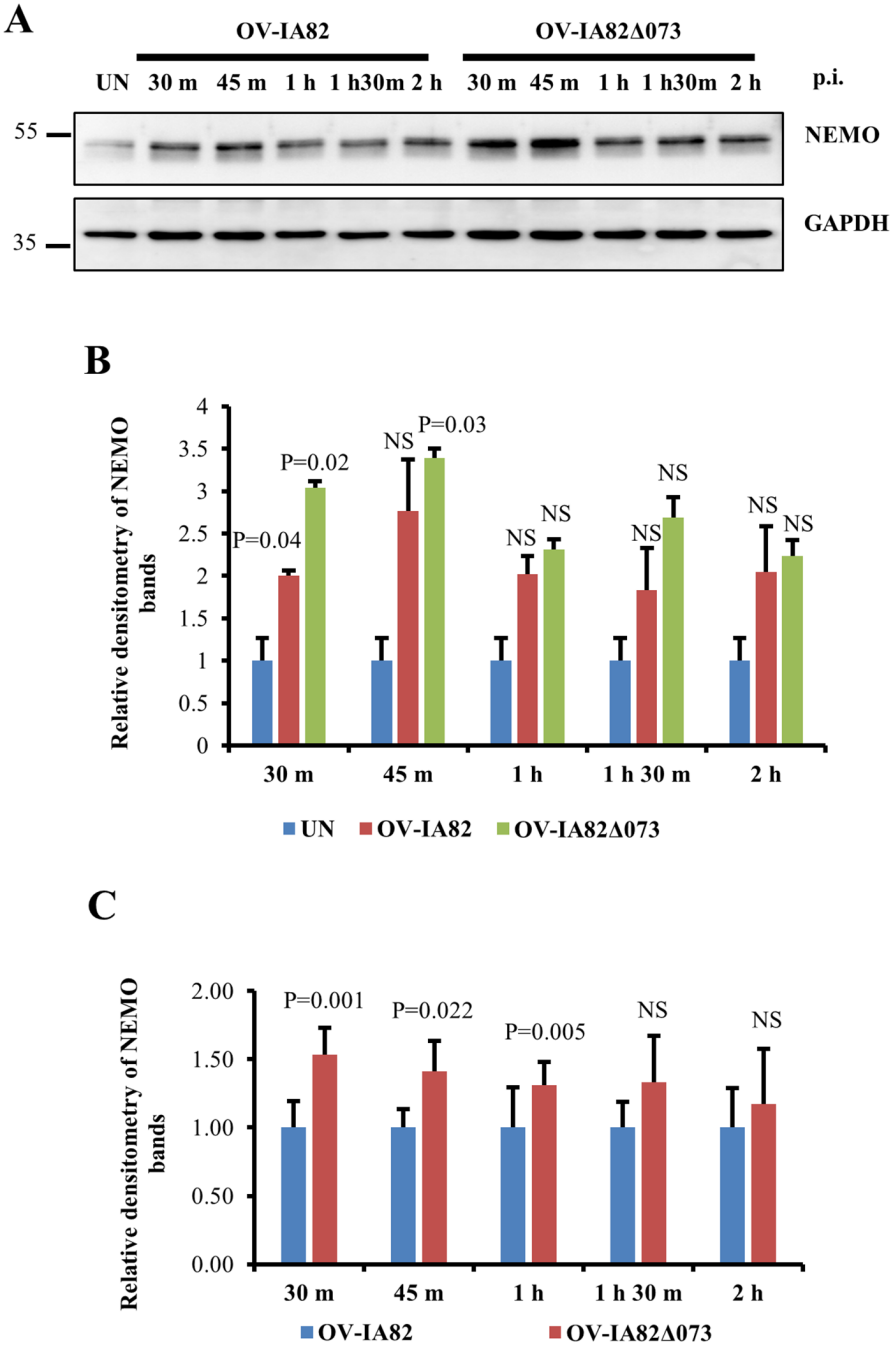
Effect of ORFV073 on NEMO protein levels during ORFV infection. (A) OFTu cells were mock infected (UN) or infected with OV-IA82 or OV-IA82Δ073 (MOI = 10) and harvested at 30 min, 45 min, 1 h, 1 h 30 min and 2 h p.i. Cytoplasmic extracts (50 μg) were resolved by SDS-PAGE, blotted to nitrocellulose membranes and probed with antibodies against NEMO and GAPDH. Densitometry of NEMO bands shown in B and C are normalized to the loading control GAPDH. (B) Fold changes are shown relative to uninfected control and results are mean values of two independent experiments (*, *P<0*.*05*). (C) Fold changes are shown relative to OV-IA82 and results are mean values of seven independent experiments (*, *P<0*.*05*)

To investigate the role of ORFV073 in NF-κB inhibition, OFTu cells were infected with OV-IA82, OV-IA82Δ073 or OV-IA82RV073^Flag^ for 30 min or 1 h, and phosphorylation of IKKα/β, IκBα and NF-κB-p65 was assessed by Western blot. Infection by OV-IA82Δ073 led to marked and early phosphorylation of IKKα/β (Ser176/180), IκBα (Ser32/36) and NF-κB-p65 (Ser536) (Fig 5A). Densitometric analysis showed that relative fold increases of phosphorylated forms in OV-IA82Δ073-infected cells were 101.7 and 123.7 for IKKα/β (Fig 5B), 54.2 and 33.7 for IκBα (Fig 5C), and 5.5 and 2.5 for NF-κB-p65 (Fig 5D), at 30 min and 1 h p.i., respectively. To assess the effect of ORFV073 on NEMO, OFTu cells were mock infected or infected with OV-IA82 or OV-IA82Δ073 for 30 min, 45 min, 1 h, 1 h 30 min and 2 h, and expression of NEMO was assessed by Western blot. Virus infection resulted in a significant increase in NEMO levels in wild-type virus-infected cells at 30 min (2.0 fold) and OV-IA82Δ073 infected cells at 30 min (3.04 fold), and 1 h p.i. (3.39 fold) compared to mock infected cells (Fig 6A and 6B). However, NEMO levels in OV-IA82Δ073 infected cells were significantly higher at 30 min (1.53 fold), 45 min (1.41 fold) and 1 h (1.31 fold) than those observed in wild-type virus-infected cells (Fig 6A and 6C). Together, results indicate that ORFV073 prevents NF-κB activation early in infection by inhibiting activation of the IKK complex. This is likely the result of a ORFV073-dependent event that leads to reduced accumulation of NEMO in wild-type virus-infected cells compared to levels found in OV-IA82Δ073 infected cells.

### ORFV073 is a virion protein and its early NF-κB inhibitory activity does not involve *de novo* viral protein synthesis

**Fig 7:**
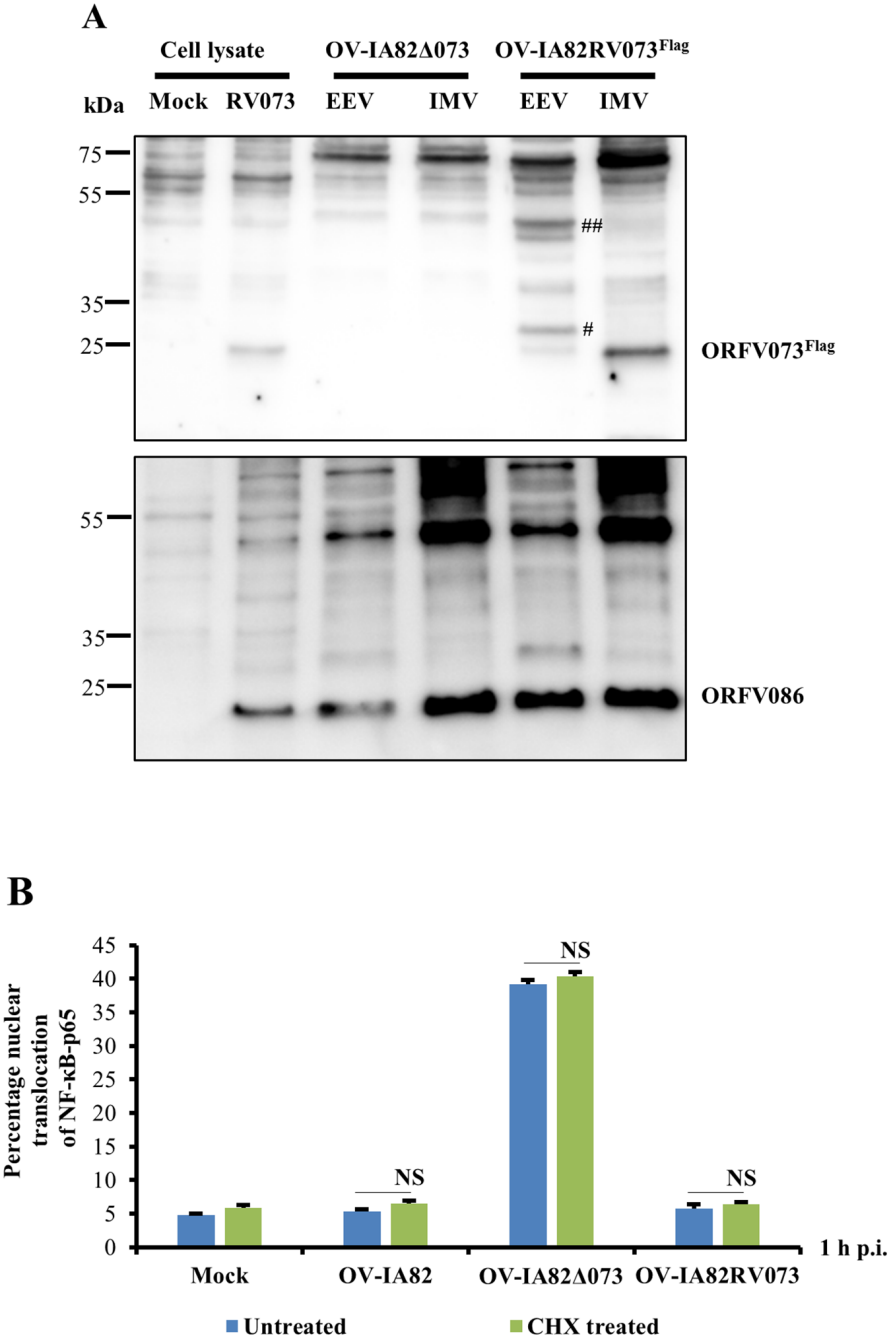
(A) Detection of ORFV073 in virions. OFTu cells were infected with OV-IA82Δ073 or OV-IA82RV073^Flag^ and harvested at 90–95% cytopathic effect. Supernatants and pellets from infected cultures were purified for extracellular enveloped virus (EEV) and intracellular mature virus (IMV) as described in Material and Methods. Whole cell lysates (10 μg) from mock and OV-IA82RV073^Flag^ infected cells (MOI = 10) (24 h p.i) and purified OV-IA82Δ073 and OV-IA82RV073^Flag^ virion proteins (10 μg) were resolved by SDS-PAGE, blotted to the nitrocellulose membrane and probed with antibodies against flag or structural protein ORFV086. # and ## denote 30 kDa and a doublet 40 kDa ORFV073 bands in EEV fraction, respectively. (B) Effect of translation inhibition on ORFV073-mediated block of NF-κB-p65 nuclear translocation. OFTu cells were pre-treated with the protein synthesis inhibitor cycloheximide (CHX) (50 μg/ml) for 30 min and then mock infected or infected with OV-IA82, OV-IA82Δ073 or OV-IA82RV073^Flag^ (MOI = 10) in presence of CHX (50 μg/ml). Cells were fixed at 1 h p.i., incubated with antibody against NF-κB-p65, stained with Alexa Fluor 488-labeled antibody and DAPI, and examined by confocal microscopy. Cells (n = approximately 300) expressing nuclear NF-κB-p65 in each group were counted and results depicted as mean percentage of cells containing nuclear NF-κB-p65 over two independent experiments (*, *P<0*.*05*). Result shown in (A) are representative of six independent experiments.

The early inhibitory effect of ORFV073 on NF-κB signaling is at variance with it being expressed at late times p.i. This observation, together with ORFV073 staining small circular to ovoid structures in infected cells (Fig 2B and S2A Fig) raised the possibility that ORFV073 may be a virion component available during and/or immediately after virus entry. To examine this possibility, extracellular enveloped virus (EEV) and intracellular mature virus (IMV) were purified from OFTu cells infected with OV-IA82RV073^Flag^. Western blot analysis showed a major band with a size corresponding to ORFV073-3xflag predicted molecular weight (approximately 25 kDa) in the IMV fraction and a noticeably weaker band in the EEV fraction which might represent possible contamination with IMV. Higher molecular weight forms of ORFV073 of approximately 30 kDa (observed in two of six independent experiments) and a doublet of 40 kDa (observed in all six independent experiments) were detected in the EEV fraction. These ORFV073 specific bands were not observed in western blots of OV-IA82Δ073 virions or uninfected cell lysates (Fig 7A, top panel). Higher molecular weight forms of ORFV073 in EEV suggest possible covalent modification of virion-incorporated ORFV073 during particle maturation and morphogenesis. As a control, the virion core protein ORFV086 was detected as a predominant 21 kDa band together with previously described higher molecular weight forms in both EEV and IMV fractions [42] (Fig 7A, bottom panel).

To assess whether early inhibition of NF-κB-p65 nuclear translocation by ORFV073 involves *de novo* viral protein synthesis in the infected cells, OFTu cells were pre-treated with the protein synthesis inhibitor cycloheximide (CHX) for 30 min followed by infection with OV-IA82, OV-IA82Δ073 or OV-IA82RV073^Flag^ for 1 h in presence of the drug. Under these conditions expression of p53, a cellular protein with short half-life, was inhibited (S3 Fig). NF-κB-p65 nuclear translocation was inhibited in both OV-IA82 and OV-IA82RV073^Flag^ -infected cells regardless of CHX treatment (Fig 7B and S4 Fig). Together, these results indicate that ORFV073 is a virion component responsible for early inhibition of NF-κB signaling.

### ORFV073 alone is sufficient for inhibiting TNFα-induced nuclear translocation of NF-κB-p65 in non-infected cells

**Fig 8:**
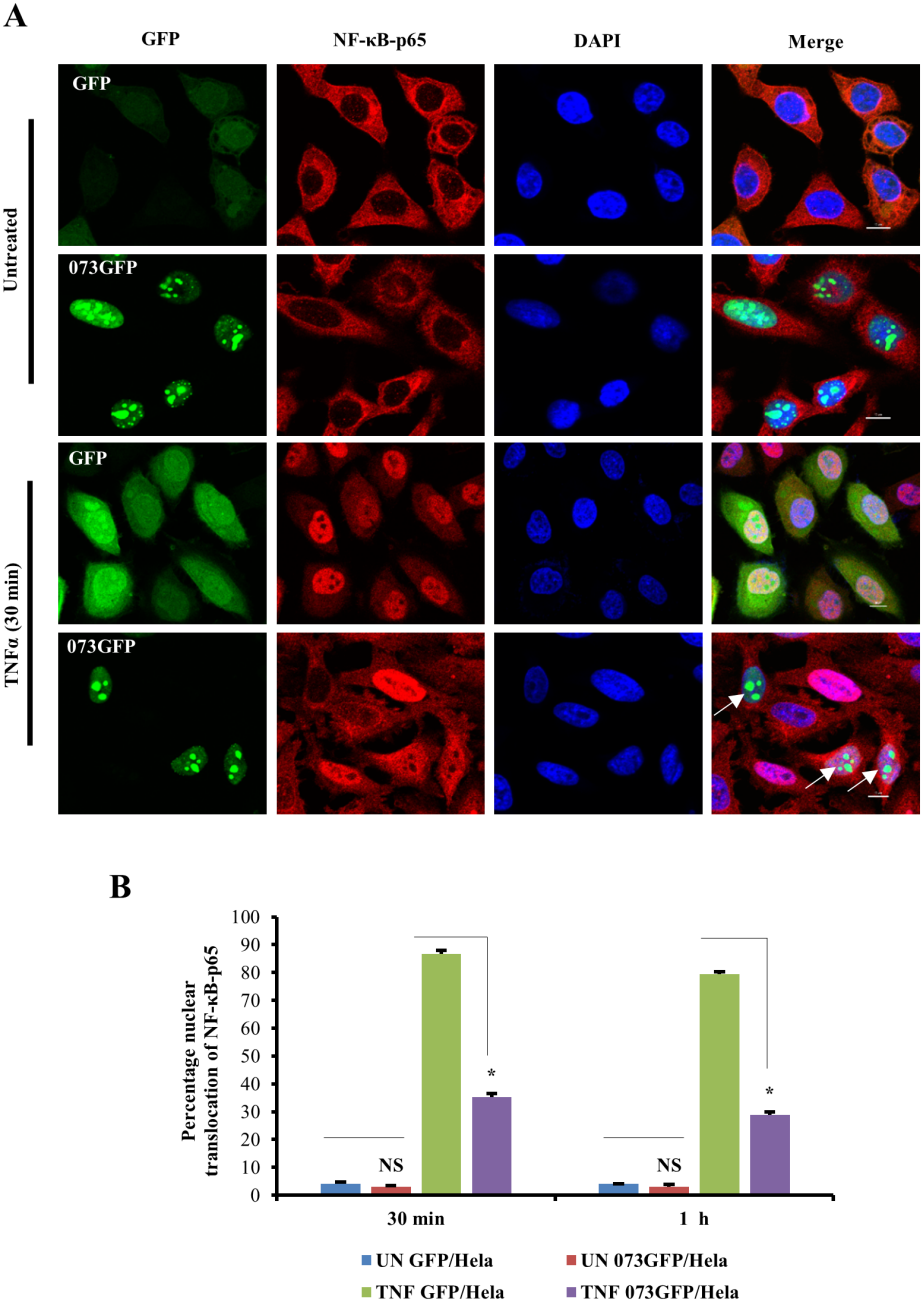
Effect of ORFV073 expression on TNFα-induced NF-κB-p65 nuclear translocation. (A) HeLa cells stably expressing GFP (GFP/HeLa) or ORFV073-GFP fusion protein (073GFP/HeLa) were treated with TNFα (20 ng/ml) and fixed 30 min or 1 h after treatment. Cells were sequentially incubated with anti-NF-κB-p65 and Alexa Fluor 594-labeled antibodies, and stained with DAPI. Shown are representative results of two independent experiments after 30 min TNFα treatment. GFP and 073GFP, Green; NF-κB-p65, red; DAPI, blue. Arrows indicate ORFV073-expressing cells with absence or reduced nuclear NF-κB-p65. (B) Cells (n = approximately 200) from randomly selected fields were scored for nuclear NF-κB-p65 and results are depicted as mean percentage of GFP/073GFP expressing cells containing nuclear NF-κB-p65 at 30 min and 1 h post TNFα induction over two independent experiments (*, P<0.05).

The effect of ORFV073 in TNFα induced nuclear translocation of NF-κB-p65 was assessed by immunofluorescence in HeLa cells stably expressing GFP (GFP/HeLa) or ORFV073GFP fusion protein (073GFP/HeLa). Upon TNFα induction, ORFV073GFP-expressing cells exhibited significantly reduced nuclear translocation of NF-κB-p65 (35.2 and 28.7%) compared to control cells expressing GFP alone (86.6 and 79.3%) at 30 min and 1 h after TNFα induction, respectively (Fig 8A and 8B, *P*<0.05). Thus, in the absence of any other viral protein, ORFV073 is able to inhibit TNFα-induced NF-κB signaling.

### ORFV073 inhibits TNFα-induced phosphorylation of IKKα/β, IκBα and NF-κB-p65 in non-infected cells

**Fig 9:**
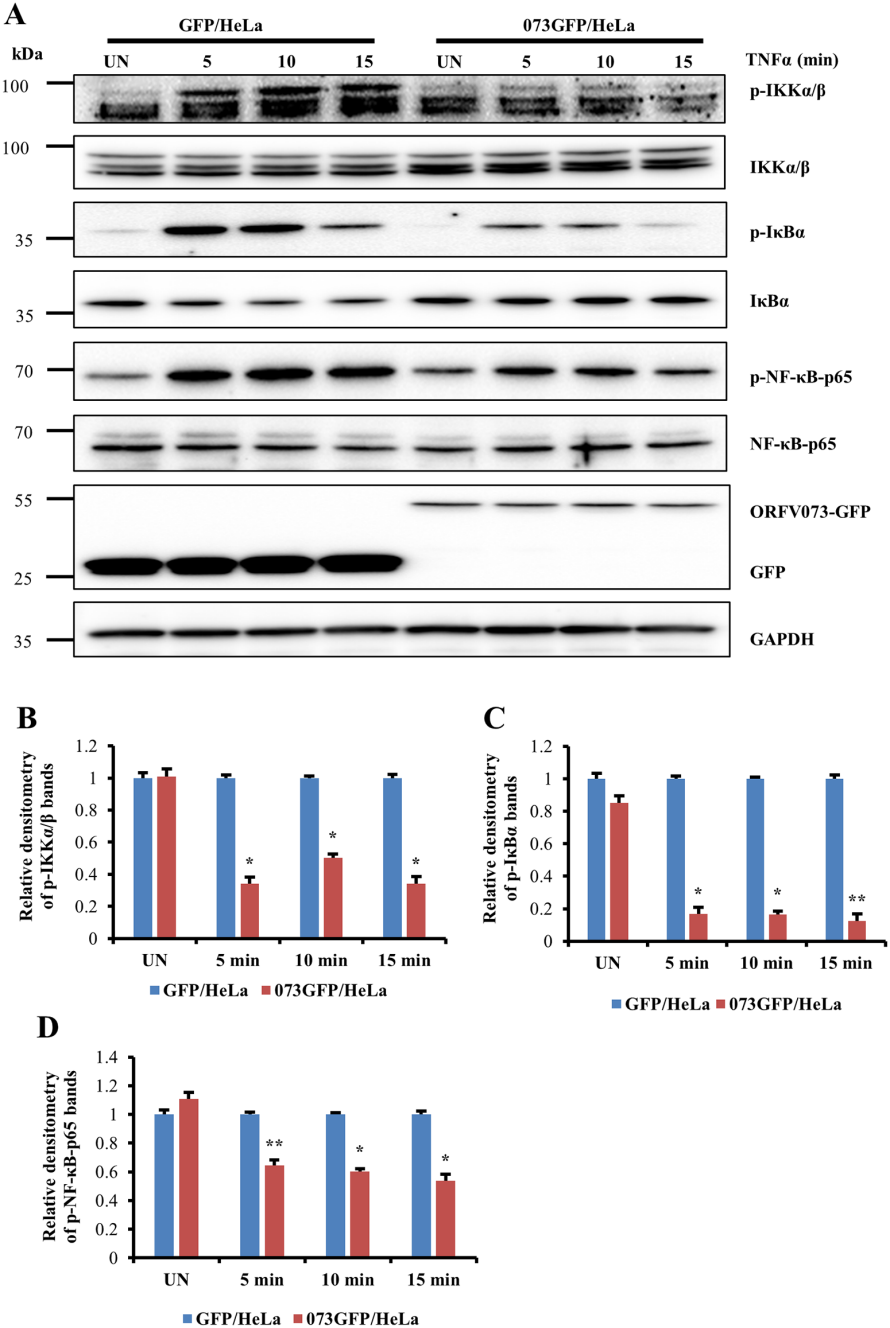
Effect of ORFV073 expression on TNFα-induced phosphorylation of IKKα/β, IκBα and NF-κB-p65. (A) HeLa cells stably expressing GFP (GFP/HeLa) or ORFV073-GFP (073GFP/HeLa) were treated with TNFα (20 ng/ml) and harvested at 5, 10 and 15 min post treatment. Whole cell protein extracts (50 μg) were resolved by SDS-PAGE, blotted to nitrocellulose membranes and probed with antibodies against phospho or total IKKα/β, IκBα and NF-κB-p65, and GAPDH and GFP. (B-D) Densitometry of p-IKKα/β, p- IκBα and p-NF-κB-p65 bands, respectively are normalized to the loading control GAPDH. Relative fold inductions across treatments in B-D are shown relative to GFP/HELA (*, *P<0*.*05* and **, *P<0*.*01*). Results are mean values of three independent experiments.

The effect of ORFV073 in TNFα induced activation of NF-κB-p65 was further investigated by examining phosphorylation of IKKα/β (Ser176/180), IκBα (Ser32/36) and NF-κB-p65 (Ser536) in HeLa cells stably expressing GFP or ORFV073GFP fusion. ORFV073 expression markedly reduced the TNFα induced phosphorylation of IKKα/β (65.7, 49.6 and 65.9%), IκBα (83, 83.4 and 87.4%) and NF-κB-p65 (35.7, 39.8 and 46%) in cells expressing 073GFP compared to control GFP expressing cells at 5, 10 and 15 min after TNFα induction, respectively (Fig 9A, 9B, 9C and 9D, *P*<0.05). While constant levels of IKKα/β, NF-κB-p65 and GAPDH were observed in mock and TNFα-treated cells, reduced levels of total IκBα were noted in ORFV073GFP cells following TNFα treatment, likely due to proteasomal degradation of IκBα following its phosphorylation. Together, results indicate that ORFV073 inhibits both virus infection-and TNFα-induced NF-κB-p65 activation by preventing activation of the IKK complex.

### ORFV073 interacts with NEMO in NF-κB signaling pathway

**Fig 10:**
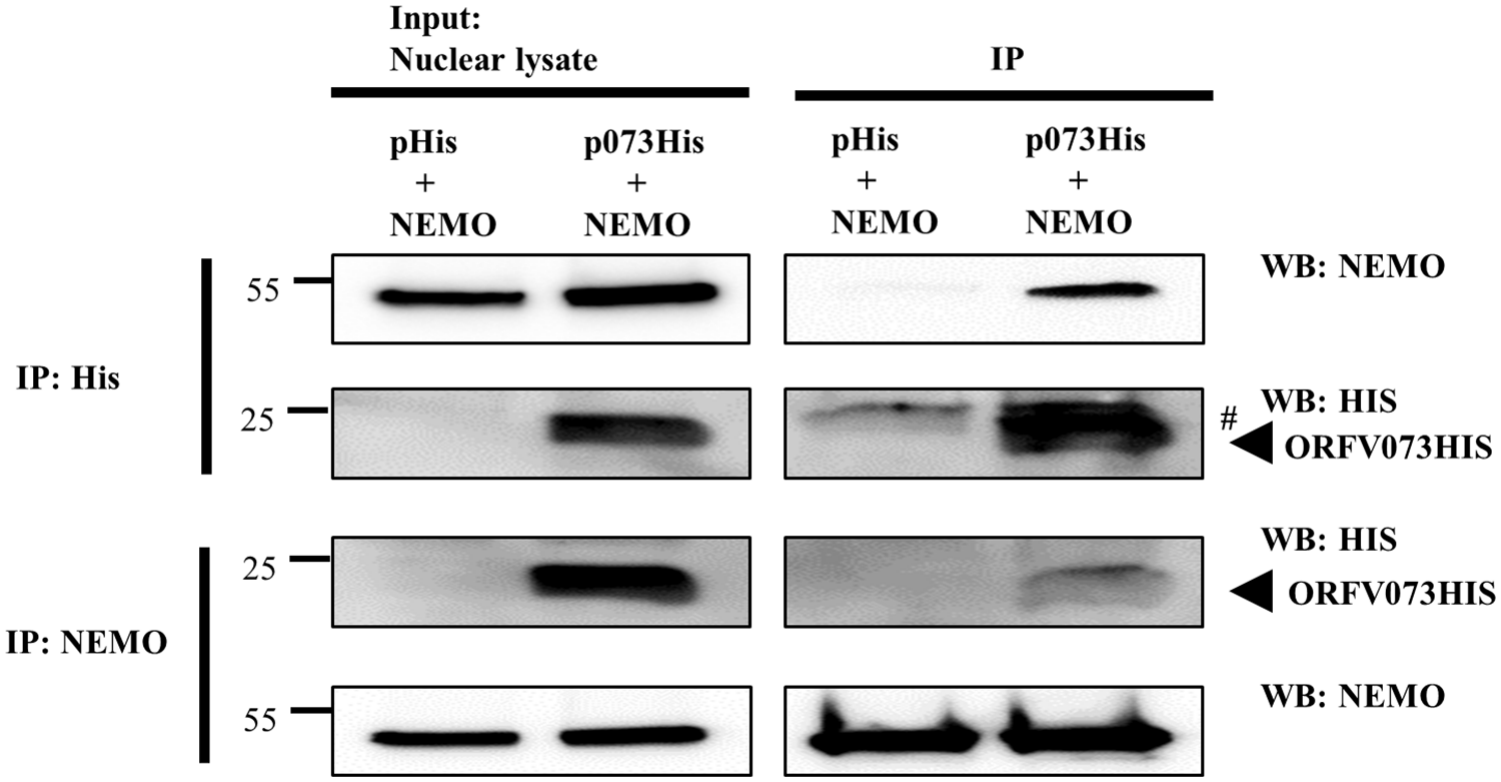
Co-immunoprecipitation of ORFV073 with NEMO. OFTu cells co-transfected with plasmids pcDNA3.1-NEMO, pcDNA/V5-His (control), or pcDNA3.1-NEMO and pcDNA/V5-073His (ORFV073-His) were harvested at 24 h post transfection and nuclear extracts prepared as described in Material and Methods. Nuclear lysates (left) and extracts immunoprecipitated with anti-His (upper right) or anti-NEMO (lower right) antibodies were examined by SDS-PAGE-Western blotting with antibodies directed against proteins indicated on the right. # denote light chain of the IgG antibody. Results are representative of three independent experiments.

Results above demonstrated that ORFV073 functions at or upstream of IKK complex in NF-κB signaling pathway. To examine the potential mechanism(s) underlying ORFV073 function, reciprocal co-immunoprecipitation of ORFV073 with various mediators of the TNFα-induced NF-κB signaling pathway was performed. OFTu cells were co-transfected with control plasmid or pORFV073-His together with pNEMO, pRIPK1, or pTRAF6. Cells were harvested 24 h post-transfection and nuclear extracts obtained as described in Material and Methods. Reciprocal interaction was observed between ORFV073 and NEMO following either anti-His or anti-NEMO antibody pull downs (Fig 10). Reciprocal co-immunoprecipitation of ORFV073 with RIPK1 and TRAF6 were not observed. These results show that ORFV073 interacts with NEMO, the regulatory subunit of the IKK complex. Interaction of ORFV073 with NEMO in uninfected cells and elevated levels of NEMO in cells infected with OV-IA82Δ073 early during infection suggest that ORFV073 interferes with assembly and/or activation of the IKK complex thus affecting subsequent activation of NF-κB signaling.

### ORFV073 contributes to ORFV virulence in the natural host

**Fig 11:**
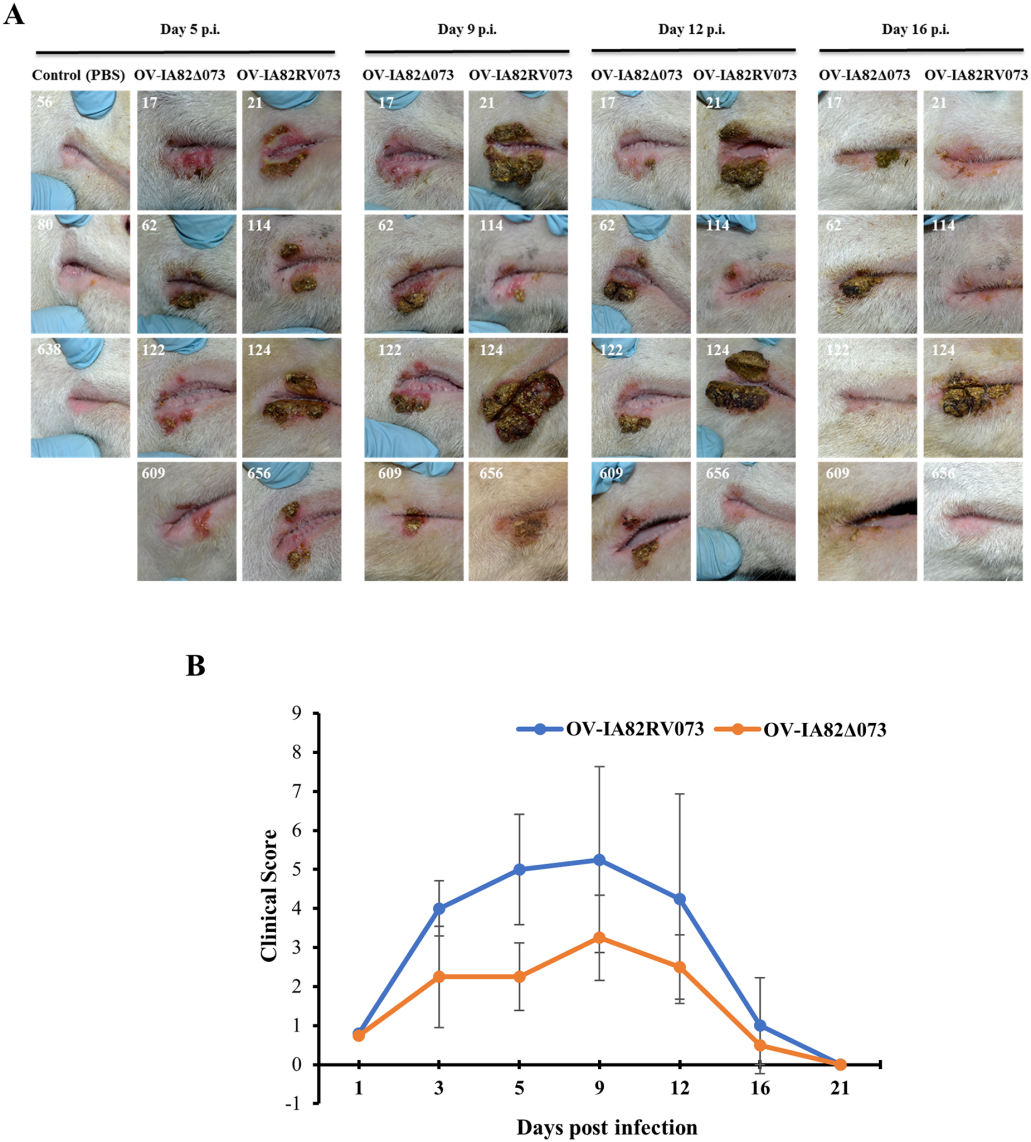
Effect of ORFV073 on virus virulence and pathogenesis in sheep. (A) Clinical course of orf in sheep infected with OV-IA82Δ073 and OV-IA82RV073^Flag^ viruses. Sheep were inoculated in the right labial commissure with OV-IA82Δ073 (sheep # 17, 62, 122, and 609), OV-IA82RV073^Flag^ (sheep 21, 114, 124 and 656) or PBS (control; sheep 56, 80, 638). Results are shown for 5, 9, 12, and 16 days p.i. (B) Clinical scores were obtained based on the extent of erythema, development of papules and pustules, and deposition of scabby tissue at scarification lines (see Materials and methods). The group mean total clinical scores are plotted against days post-infection.

The effect of ORFV073 in virus virulence was investigated in sheep, a natural ORFV host. Animals were inoculated with OV-IA82Δ073 (n = 4), OV-IA82RV073^Flag^ (n = 4) or PBS (control group, n = 3) in the right labial commissure and the inner side of the thighs, and disease course was monitored for 21 days. All virus-inoculated animals developed clinical orf (Fig 11A). However, clinical disease was less severe in sheep infected with OV-IA82Δ073 (Fig 11B). No significant differences in disease onset and time to lesion resolution between animal groups were observed. By day 5 p.i., lesions in all four OV-IA82RV073^Flag^ -infected sheep exhibited scabby tissue deposition and pustules at the lesion margins. In OV-IA82Δ073 -infected sheep, however, pustule development was not observed and deposition of scabby tissue was seen in only one animal at this time point (sheep #62; Fig 11A, 5 dpi). Lesions in two OV-IA82RV073^Flag^ -infected sheep continued to evolve by further scabby tissue deposition during the following week (sheep 21 and 124), while scabs in the other two animals were shed leaving pustules exposed. In contrast, changes in sheep inoculated with mutant virus progressed modestly and a clinical pustular stage was never observed (Fig 11A, Day 9 p.i.). Lesions started to regress by day 12 p.i., with one animal per group exhibiting scabby lesions at day 16 p.i. (sheep 62 and 124), and clinical resolution was complete in all virus-infected sheep by day 21 p.i.

**Fig 12:**
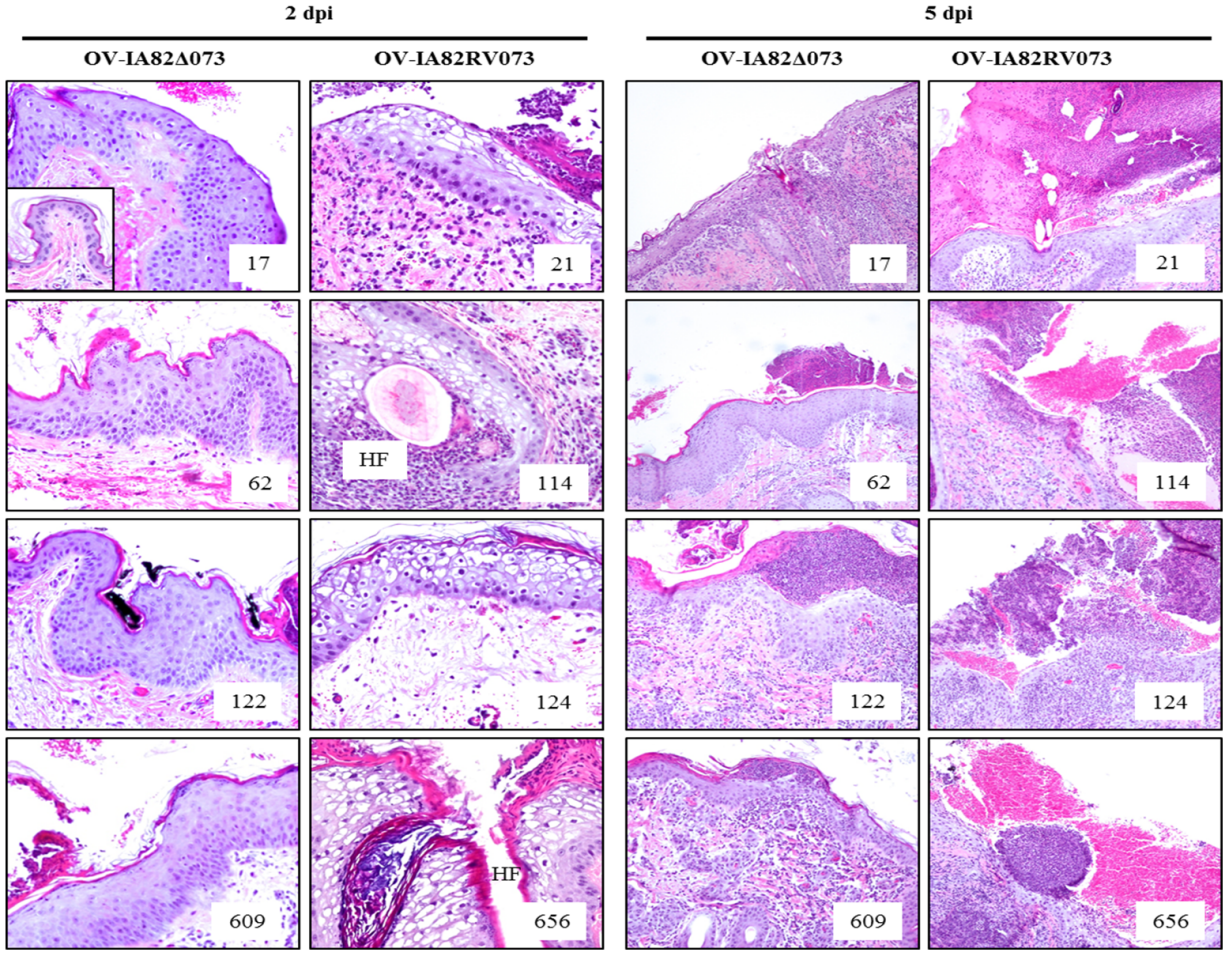
Histological changes in the skin of the inner side of thighs following inoculation with OV-IA82Δ073 (sheep 17, 62, 122, and 609) and OV-IA82RV073 (sheep 21, 114, 124, and 656). Results are shown for days 2 and 5 p.i. Inset in top left panel shows normal skin at the inoculation site (uninfected control). HF, hair follicle. H&E, 2 dpi and ctrl, X200; 5 dpi, X100.

Punch biopsies were collected from inoculation sites in the thighs at various times post-infection and processed for histology. By 2 dpi, skin samples from all animals showed epidermal hyperplasia, active re-epithelialization, and various degrees of inflammatory cell infiltration. All OV-IA82RV073^Flag^ -infected sheep showed foci of ballooned degenerated keratinocytes, a morphological indication of advanced virus replication. In contrast, none of the OV-IA82Δ073 -infected sheep exhibited ballooned degeneration by this time. (Fig 12, left panels). By day 5 p.i., with the exception of sheep 62, samples from all infected animals exhibited ballooning degeneration of keratinocytes. Congruent with the gross pathology, OV-IA82RV073^Flag^ -infected sheep samples showed large, often broken and hemorrhagic pustules. In contrast, lesions in OV-IA82Δ073-infected sheep contained small, intact micropustules contained by a mildly hyperkeratotic stratum corneum (Fig 12, right panels). These pustules never developed further beyond this stage. Data indicate that infection of sheep with OV-IA82Δ073, a virus lacking ORFV073, resulted in delayed infection of keratinocytes and absence of a clinical pustular stage.

## Discussion

NF-κB is a key regulator of early host responses against pathogens, playing a critical role in inflammation and integrating many cellular processes including cell proliferation, differentiation, and survival [9,10]. The parapoxvirus ORFV has evolved multiple strategies to counteract activation of the NF-κB signaling pathway, with encoded NF-κB inhibitors targeting both cytoplasmic and nuclear events leading to NF-κB activation [36–38]. Here, we describe a ORFV virion protein, ORFV073, that inhibits activation of the IKK complex and subsequent NF-κB signaling at very early times post-infection.

Parapoxviral genes involved in host range, immune modulation/evasion and virulence largely map to the terminal genomic regions [40,44]. Somewhat surprisingly, *ORFV073*, is located approximately in the center of the central conserved region of the genome, between two highly conserved poxviral genes (ORFV072, which encodes for a transcription termination factor, and ORFV074, which encodes for the small subunit of the mRNA capping enzyme). ORFV073 is highly conserved among the ORFV isolates and, while less similar to orthologs in other parapoxviruses, it still exhibits a higher degree of conservation than that observed for other known parapoxviral host range and immune evasion genes [40]. ORFV073 genomic location and its high degree of conservation may suggest the overall significance of this nonessential gene for viral perpetuation and transmission under selective pressures operating in nature. Interestingly, the finding of a 50 aa region in ORFV073 with homology to a herpesvirus protein of unknown function (mouse cytomegalovirus m170) suggests that yet unmapped ORFV073 functions may extend across virus families.

Notably, ORFV073 is a virion protein that inhibits NF-κB signaling at very early times in infected cells (≤ 30 min. p.i.) (Figs 4C, 5A, 7A and 7B). Our experiments with virus lacking ORFV073 suggest that early infection events such as virus entry and uncoating are efficiently sensed by PRRs, leading to NF-κB activation. Recently, tumor necrosis factor receptor (TNFR)-associated factor 2 (TRAF2) was reported to be involved in VACV fast entry via plasma membrane fusion [45]. TRAF2 functions downstream of TNFR1 and TNFR2 mediating activation of both canonical and non-canonical NF-κB signaling pathways [46]. If TRAF2 is activated in some manner during virus entry, subsequent activation of intracellular signaling pathways, including the NF-κB pathway, would be the expected outcomes. In the context of wild-type ORFV infection, virion-associated ORFV073 is immediately available to interfere with any potential TRAF2-induced NF-κB activation by inhibiting IKK activation possibly by interaction with NEMO. In contrast, a virus lacking ORFV073 in the virion, such as OV-IA82Δ073 described here, would be unable to block NF-κB early activation and nuclear translocation of NF-κB-p65. While other scenarios are also possible, results here illustrate the importance of preventing NF-κB activation early in infection.

In the context of the virus-infected cell, relatively few poxviral NF-κB inhibitors with clearly defined early functions have been described. VACV K1L protein was shown to prevent degradation of IκBα between 2 and 3 h p.i. in infected cells [25]. Similarly, VACV B14 was shown to reduce phosphorylation of IκBα at 2 and 4 h p.i [47] and VACV M2L was shown to inhibit phosphorylation of extracellular signal-regulated kinase 1 and 2 (ERK1/2) at 2 h p.i. and subsequent activation of NF-κB signaling [24]. Likewise, ORFV ORFV121 and ORFV002 were shown to inhibit NF-κB-p65 phosphorylation and acetylation, respectively, at relatively early times p.i [37,38].

ORFV073 inhibits NF-κB-p65 activation by preventing activation of IKK complex (Figs 5A and 9A). ORFV073 interaction with NEMO, the regulatory subunit of the IKK complex, likely underlies this inhibition (Fig 10). The early inhibition of IKK complex in wild-type ORFV-infected cells, is coincident with the reduced levels of NEMO in wild-type virus-infected cells compared to levels observed in OV-IA82Δ073-infected cells during the first hour p.i., suggesting that altered NEMO protein stability and/or trafficking might occur in the presence of ORFV073 (Fig 6A and 6C). Other poxviral NF-κB inhibitors are reported to specifically target the IKK complex, the bottleneck for most NF-κB-activating signals [19]. ORFV ORFV024 was shown to inhibit activation of IKK complex by preventing phosphorylation of IKK kinase [36]. VACV B14 and N1L were shown to interact with IKKβ and multiple components of IKK complex, respectively inhibiting subsequent activation of IKK complex [23,47]. Similarly, MCV MC159 and MC160, were shown to interact with NEMO preventing IKKβ activation and induce degradation of IKKα, respectively [33,34].

ORFV073 is a late viral protein found predominantly in the nucleus of infected cells at 16 to 24 h p.i. (Fig 2A and 2B). Late expression of ORFV073 in the viral replicative cycle is consistent with it being a virion component and functioning early in the next round of infection; however, the predominant nuclear localization of the protein at late times p.i. suggests it may have additional functions, related or unrelated to the NF-κB signaling pathway. Other poxviral NF-κB inhibitors with nuclear functions have been described. For example, parapoxviral ORFV002 is a nuclear inhibitor of the NF-κB signaling pathway that affects NF-κB-p65-mediated transcription [37]. The myxoma virus virulence factor M150R colocalized with NF-κB-p65 in the nucleus of TNFα-stimulated cells suggesting a potential role in regulation of the NF-κB signaling pathway; however, its effect on NF-κB-mediated gene transcription has not been demonstrated [48]. A nuclear function leading to decreased NF-κB-mediated gene expression was reported for VACV, but the actual viral protein(s) and mechanism(s) responsible for the inhibition are still unknown [49]. Recently, VACV K1 protein was shown to localize in both cytoplasm and nucleus of the cell, and prevent NF-κB-p65 acetylation [50].

Interestingly, ORFV073 interacts with NEMO in the nucleus of *ORFV073* transfected cells. In addition to the canonical and non-canonical NF-κB pathway, NEMO is also involved in the atypical NF-κB pathway [51]. In response to genotoxic stress, which conceivably could occur during later stages of virus infection, NEMO translocates to the nucleus where it undergoes ataxia telangiectasia mutated checkpoint kinase (ATM)-mediated ubiquitination. NEMO and ATM are then trafficked to the cytoplasm where they activate IKKβ which results in activation of the canonical NF-κB pathway [11,52]. Although no significant differences in NF-κB-p65 nuclear translocation were observed between wild type- and OV-IA82Δ073-infected cells at late times p.i. (Fig 4C and 4D), possible effects of ORFV073-NEMO interactions on NF-κB signaling in the nucleus cannot be excluded. Other, as yet uncharacterized, late nuclear functions for ORFV073 unrelated to the NF-κB signaling pathway are also possible.

The actual role of poxviral NF-κB inhibitors for aspects of infection biology *in vivo* remains poorly understood [18,21,36,37,53]. Here, deletion of *ORFV073* from the ORFV genome resulted in attenuation of ORFV in sheep, indicating that ORFV073 contributes to ORFV virulence in the natural host. The delayed infection of keratinocytes and absence of a clinical pustular stage in sheep infected with OV-IA82Δ073 likely reflect improved ability of the host to control the infection in the absence of ORFV073. Studies with other ORFV-encoded NF-κB inhibitors have shown that single genes either had no effect on disease pathogenesis, resulting in a wild-type disease phenotype in sheep (ORFV002, ORFV024) [36,37] or, as for ORFV121, a viral protein which binds to- and prevents nuclear translocation of NF-κB-p65, led to a markedly attenuated disease phenotype [38]. Remarkably, single gene deletions of most poxviral NF-κB inhibitors resulted in only modest effects on viral pathogenesis and virulence [22,36,37]. The multiple NF-κB inhibitors encoded by a poxvirus together with the possibility of overlapping or complementing functions may explain this observation. Alternatively, specific poxviral NF-κB inhibitors may exert only subtle and perhaps transient host range effects on specific infected cells or the infected tissue microenvironment. Regardless, the impact of these subtle changes on virus fitness in nature may be difficult to fully ascertain under experimental conditions.

To our knowledge, ORFV073 is the first poxviral NF-κB inhibitor found in virions. As early infection events are likely conserved among poxviruses [54], it is reasonable to speculate that other poxviruses encode yet to be identified virion proteins which inhibit NF-κB activation very early in infection and that early inhibition of NF-κB signaling is of greater biologic significance than currently appreciated.

## Supporting information

S1 Fig Transcriptional kinetics of *ORFV073*. Transcription kinetics of *ORFV073*, *ORFV085* (late gene control) and *ORFV127* (early gene control) was assessed during ORFV infection in OFTu cells in the presence (+) or absence (-) of AraC. Cells infected with OV-IA82 (MOI = 10) were harvested at respective times and transcription levels were determined by RT-PCR. Results are representative of two independent experiments.
(TIF)

S2 Fig Co-staining of ORFV073 with ORFV086 and endosomal marker. (A) Co-localization of ORFV073 and ORFV086 in OFTu cells mock infected or infected with revertant virus OV-IA82RV073^Flag^ (MOI = 10). Immunofluorescence and confocal microscopy was performed as described in Material and Methods. Shown are representative images from 24 h p.i. ORFV073, green; ORFV086; Magenta; DAPI, blue. Results are representative of two independent experiments. (B) Subcellular localization of ORFV073 and endosomal marker (Caveolin-1) in OFTu cells mock infected or infected with revertant virus OV-IA82RV073^Flag^ (MOI = 10). Immunofluorescence and confocal microscopy was performed as described in Material and Methods. Shown are representative images from 24 h p.i. ORFV073, green; Caveolin-1; Magenta; DAPI, blue. Results are representative of two independent experiments.
(TIF)

S3 Fig Effect of translation inhibition on expression of p53. OFTu cells mock treated or pre-treated with CHX (50 μg/ml) for 30 min were mock infected or infected with OV-IA82RV073^Flag^ (MOI = 10) in presence of CHX (50 μg/ml) and harvested at 30 min and 1 h p.i. Whole cell protein extracts (50 μg) were resolved by SDS-PAGE, and transferred to nitrocellulose membranes and probed with antibody against p53 and actin. Results are representative of two independent experiments.
(TIF)

S4 Fig Effect of translation inhibition on ORFV073-mediated inhibition of NF-κB-p65 nuclear translocation. OFTu cells mock treated or pre-treated with CHX (50 μg/ml) for 30 min were mock infected or infected with OV-IA82, OV-IA82Δ073 or OV-IA82RV073^Flag^ (MOI = 10) in presence of CHX (50 μg/ml). Cells were fixed at 1 h p.i., incubated with antibody against NF-κB-p65, stained with Alexa Fluor 488-labeled antibody and DAPI, and examined by confocal microscopy. Results are representative of two independent experiments.
(TIF)
