# Supplementary figures and images for: A parapoxviral virion protein inhibits NF-κB signaling early in infection
Source: PLoS Pathog. 2017 Aug 7;13(8):e1006561. doi: 10.1371/journal.ppat.1006561 (PMC5560748; doi:10.1371/journal.ppat.1006561)

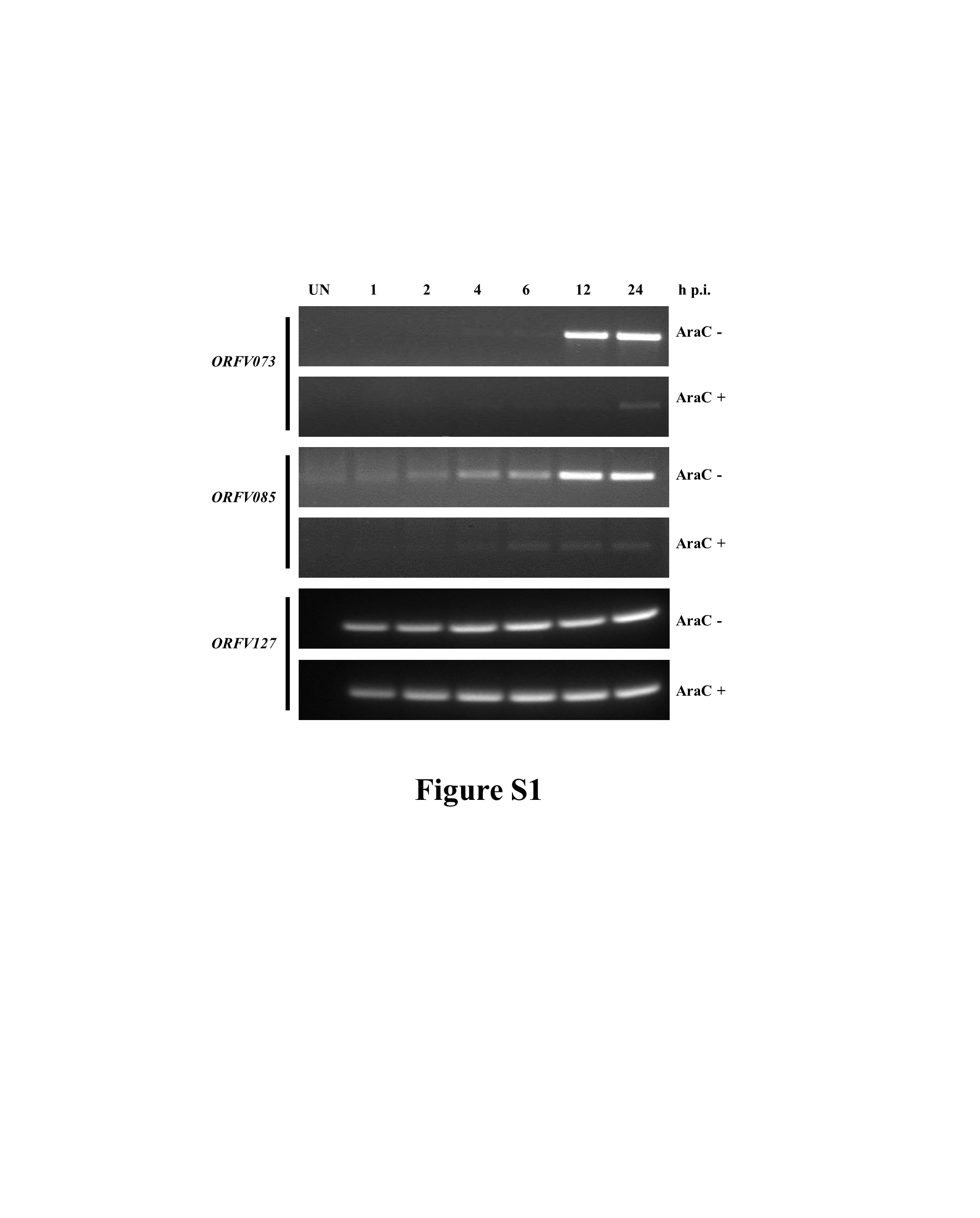

Supplement: S1 Fig — Transcription kinetics of ORFV073, ORFV085 (late gene control) and ORFV127 (early gene control) was assessed during ORFV infection in OFTu cells in the presence (+) or absence (-) of AraC. Cells infected with OV-IA82 (MOI = 10) were harvested at respective times and transcription levels were determined by RT-PCR. Results are representative of two independent experiments. (TIF) [file ppat.1006561.s001.TIF]

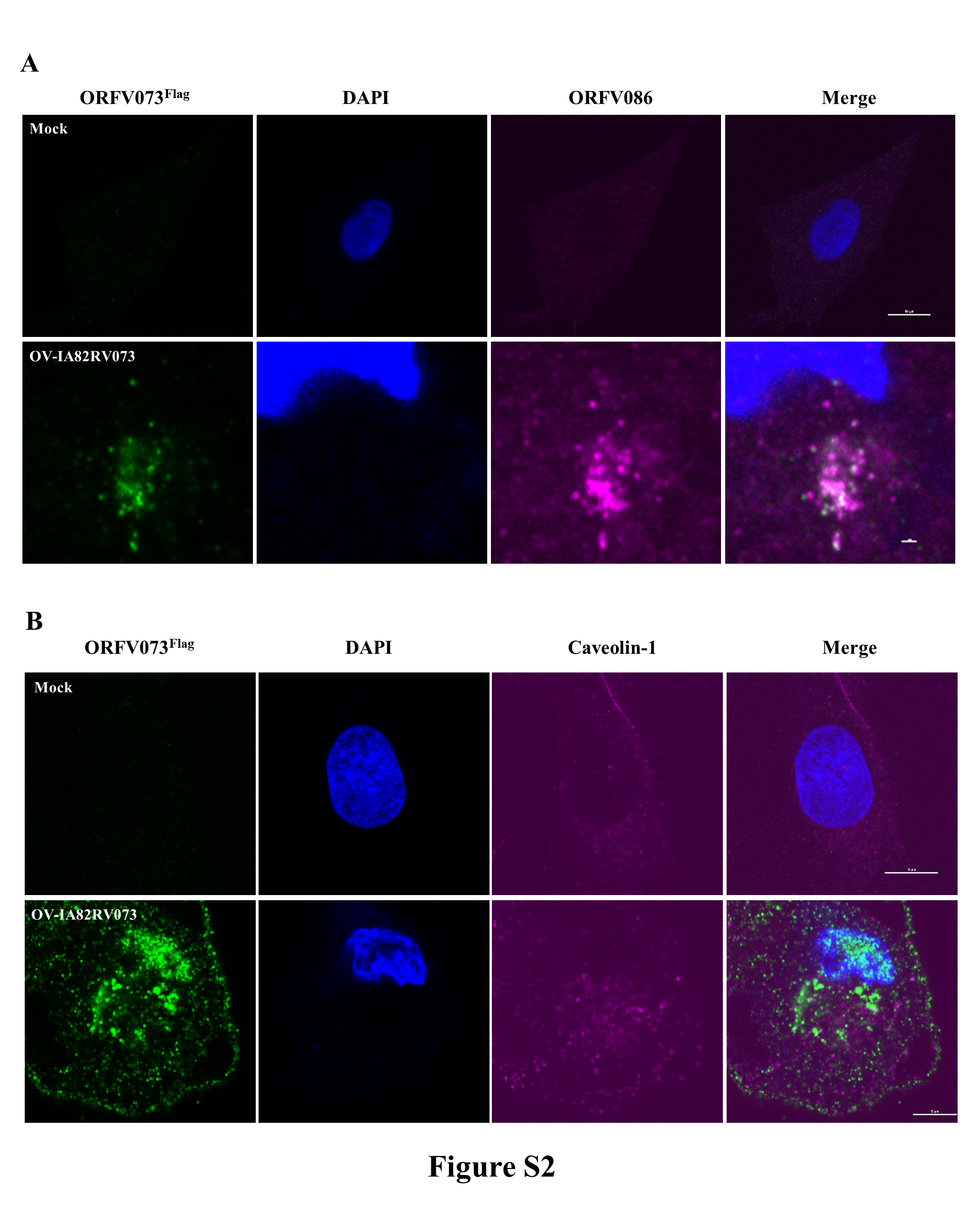

Supplement: S2 Fig — (A) Co-localization of ORFV073 and ORFV086 in OFTu cells mock infected or infected with revertant virus OV-IA82RV073Flag (MOI = 10). Immunofluorescence and confocal microscopy was performed as described in Material and Methods. Shown are representative images from 24 h p.i. ORFV073, green; ORFV086; Magenta; DAPI, blue. Results are representative of two independent experiments. (B) Subcellular localization of ORFV073 and endosomal marker (Caveolin-1) in OFTu cells mock infected or infected with revertant virus OV-IA82RV073Flag (MOI = 10). Immunofluorescence and confocal microscopy was performed as described in Material and Methods. Shown are representative images from 24 h p.i. ORFV073, green; Caveolin-1; Magenta; DAPI, blue. Results are representative of two independent experiments. (TIF) [file ppat.1006561.s002.TIF]

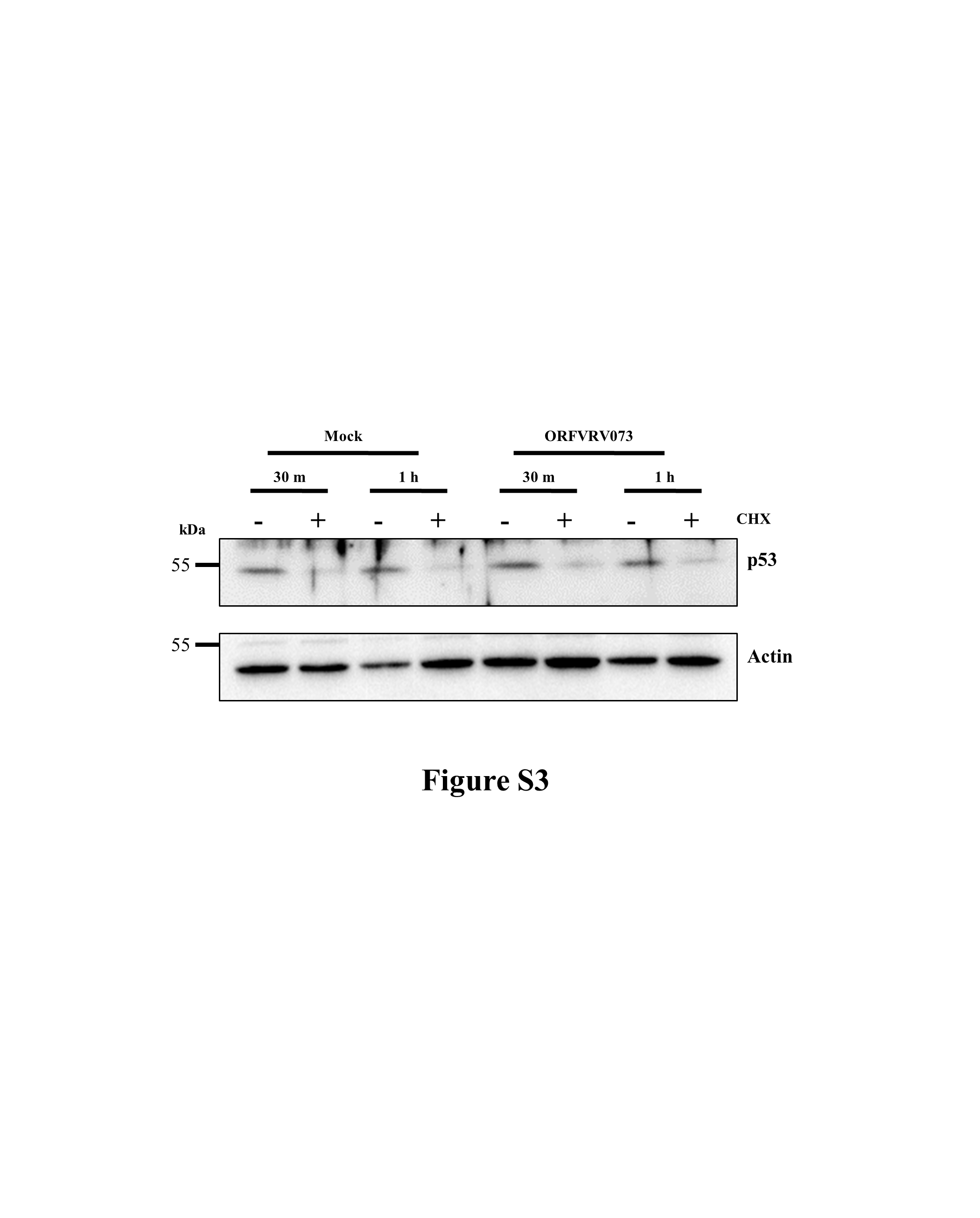

Supplement: S3 Fig — OFTu cells mock treated or pre-treated with CHX (50 μg/ml) for 30 min were mock infected or infected with OV-IA82RV073Flag (MOI = 10) in presence of CHX (50 μg/ml) and harvested at 30 min and 1 h p.i. Whole cell protein extracts (50 μg) were resolved by SDS-PAGE, and transferred to nitrocellulose membranes and probed with antibody against p53 and actin. Results are representative of two independent experiments. (TIF) [file ppat.1006561.s003.TIF]

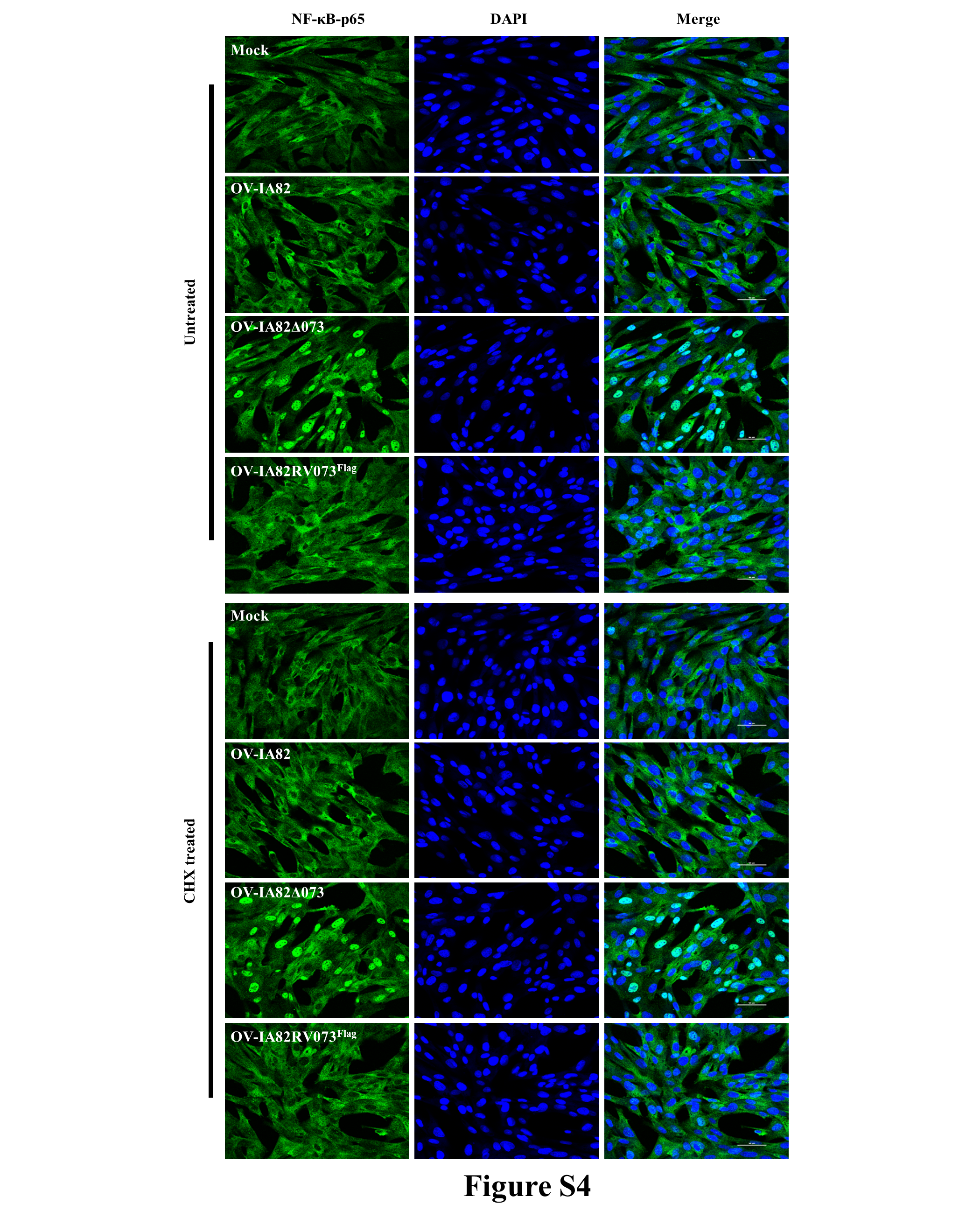

Supplement: S4 Fig — OFTu cells mock treated or pre-treated with CHX (50 μg/ml) for 30 min were mock infected or infected with OV-IA82, OV-IA82Δ073 or OV-IA82RV073Flag (MOI = 10) in presence of CHX (50 μg/ml). Cells were fixed at 1 h p.i., incubated with antibody against NF-κB-p65, stained with Alexa Fluor 488-labeled antibody and DAPI, and examined by confocal microscopy. Results are representative of two independent experiments. (TIF) [file ppat.1006561.s004.TIF]
